# The social role of C-reactive protein point-of-care testing to guide antibiotic prescription in Northern Thailand

**DOI:** 10.1016/j.socscimed.2018.02.018

**Published:** 2018-04

**Authors:** Marco J. Haenssgen, Nutcha Charoenboon, Thomas Althaus, Rachel C. Greer, Daranee Intralawan, Yoel Lubell

**Affiliations:** a Centre for Tropical Medicine and Global Health, Nuffield Department of Clinical Medicine, University of Oxford, Old Road Campus, Roosevelt Drive, Oxford OX3 7FZ, UK; b CABDyN Complexity Centre, Saïd Business School, University of Oxford, Park End Street, Oxford OX1 1HP, UK; c Green Templeton College, 43 Woodstock Road, Oxford OX2 6HG, UK; d Mahidol Oxford Tropical Medicine Research Unit (MORU), Faculty of Tropical Medicine, Mahidol University, 3/F, 60th Anniversary Chalermprakiat Building, 420/6 Rajvithi Road, Bangkok, Thailand; e Primary Care Department, Chiangrai Prachanukroh Hospital, Chiang Rai, Thailand

**Keywords:** Antimicrobial resistance, Antibiotic use, Primary care, Biomarker testing, C-reactive protein point-of-care testing, Social research, Qualitative research, Thailand

## Abstract

New and affordable point-of-care testing (POCT) solutions are hoped to guide antibiotic prescription and to help limit antimicrobial resistance (AMR)—especially in low- and middle-income countries where resource constraints often prevent extensive diagnostic testing. Anthropological and sociological research has illuminated the role and impact of rapid point-of-care malaria testing. This paper expands our knowledge about the social implications of non-malarial POCT, using the case study of a C-reactive-protein point-of-care testing (CRP POCT) clinical trial with febrile patients at primary-care-level health centres in Chiang Rai province, northern Thailand. We investigate the social role of CRP POCT through its interactions with (a) the healthcare workers who use it, (b) the patients whose routine care is affected by the test, and (c) the existing patient-health system linkages that might resonate or interfere with CRP POCT. We conduct a thematic analysis of data from 58 purposively sampled pre- and post-intervention patients and healthcare workers in August 2016 and May 2017.

We find widespread positive attitudes towards the test among patients and healthcare workers. Patients’ views are influenced by an understanding of CRP POCT as a comprehensive blood test that provides specific diagnosis and that corresponds to notions of good care. Healthcare workers use the test to support their negotiations with patients but also to legitimise ethical decisions in an increasingly restrictive antibiotic policy environment. We hypothesise that CRP POCT could entail greater patient adherence to recommended antibiotic treatment, but it could also encourage riskier health behaviour and entail potentially adverse equity implications for patients across generations and socioeconomic strata. Our empirical findings inform the clinical literature on increasingly propagated point-of-care biomarker tests to guide antibiotic prescriptions, and we contribute to the anthropological and sociological literature through a novel conceptualisation of the patient-health system interface as an activity space into which biomarker testing is introduced.

## Highlights

• Novel social research contribution to the study of antimicrobial resistance.
• Examines increasingly popular diagnostic point-of-care testing (POCT).
• Analyses POCT introduction at the patient – health system interface.
• Reveals interferences of test with patient and health worker behaviour.
• Provides evidence of un-/intended behavioural effects of point-of-care tests.

## 1 Introduction

Antibiotics procured through formal and informal channels are popularly over- and misused across high-, middle-, and low-income countries, which contributes to the development of antimicrobial resistance (AMR) and potentially to the spread of resistant bacteria across the world (Butler et al., 2009; Kumarasamy et al., 2010; Morgan et al., 2011). A range of antibiotic policies and interventions have emerged to improve clinical antibiotic prescriptions worldwide (Davey et al., 2017), including biomarker tests to inform healthcare workers’ (HCWs’) prescription decisions (Nora et al., 2017). C-reactive protein (CRP) as a biomarker of bacterial infection is one of these interventions (Lubell et al., 2015), and new point-of-care testing (POCT) solutions are hoped to become an additional tool for low- and middle-income countries (LMICs) to limit the growing problem of AMR (Do et al., 2016; Drain et al., 2014; Lubell and Althaus, 2017).

In the context of AMR, host-response biomarkers such as CRP aim at identifying patients requiring an antibiotic while helping to rule out antibiotic prescriptions when an illness is caused by a mild bacterial infection or by a viral infection (for which antibiotic treatment would be ineffectual) (Aabenhus et al., 2014, 6). As a diagnostic tool, CRP POCT can therefore indicate whether an antibiotic might be needed, but it does not provide information about which specific antibiotic would be best suited for treating the patient (Lubell et al., 2015). A review by Aabenhus et al. (2014, 6) also lists a range of potential disadvantages of such point-of-care tests, including “suboptimal use of time, costs, handling errors, patient dissatisfaction and false negative values that can lead to lack of necessary antibiotic treatments or false positive values that may increase inappropriate antibiotic use.” The rationale behind their introduction is therefore not that they are perfect diagnostic devices, but that they can aid and support clinical diagnosis in a resource-poor environment of high or ill-targeted antibiotic use until superior and affordable pathogen-specific tests become available (Lubell and Althaus, 2017).

While the recent sociological and anthropological literature on rapid diagnostic testing for malaria has expanded our understanding of POCT technologies in LMIC health systems, very little experience exists with non-malaria POCT. This study examines the case of Chiang Rai in northern Thailand, where we collected qualitative data from 58 fever patients and healthcare workers alongside a clinical trial of CRP POCT to reduce antibiotic prescriptions. Our exploratory research question is, “*What role does CRP POCT play in primary-care-level antibiotic prescription within an existing system of practices at the patient-health system interface?*”, for which we draw on a novel analytical framework that borrows arguments from the treatment-seeking behaviour, street-level bureaucracy, and actor-network theory literature. We respond thereby to recent calls from both social scientists (e.g. Chandler et al., 2016) and medical researchers (e.g. Zellweger et al., 2017) for a greater involvement of the social sciences in antimicrobial resistance research.

Our analysis will demonstrate that CRP POCT is introduced into a context of existing antibiotic-related practices. The test becomes re-defined in relation to these practices, but it also alters them. Patients’ views are thus influenced by an understanding of CRP POCT as a comprehensive blood test that provides specific diagnoses and that corresponds to notions of good care. Healthcare workers use the test to support their negotiations with patients but also to legitimise ethical decisions in an increasingly restrictive antibiotic policy environment. Commonly found positive attitudes, reassurance, and trust may therefore support the tests’ primary objective to reduce unnecessary antibiotic prescriptions, but they can also mask the unintended social consequences of altering the patient-health system relationship.

## 2 Literature and framework

### 2.1 Related literature

The clinical literature has been considering the role of biomarkers such as CRP or procalcitonin to inform and guide antibiotic prescriptions in hospitals and primary care settings in high- as well as low- and middle-income contexts (Hildenwall et al., 2017; Lubell et al., 2015; Nora et al., 2017). This has led to the development of interventions using biomarker point-of-care tests like CRP to target and reduce antibiotic prescriptions for unspecified fevers and acute respiratory infections (Aabenhus and Jensen, 2016; Aabenhus et al., 2014; Davey et al., 2017). The growing number of clinical trials primarily from high-income contexts thereby indicates moderate effectiveness of CRP testing in reducing clinically unnecessary antibiotic prescriptions (Aabenhus et al., 2014; Cals et al., 2010; Cooke et al., 2015; Do et al., 2016), making these point-of-care tests a promising and economical addition to a necessarily broad portfolio of strategies to address antimicrobial resistance in LMIC settings with resource constraints and scarce laboratory capacity (Aabenhus and Jensen, 2016; Drain et al., 2014; Lubell and Althaus, 2017). A small number of clinically oriented qualitative and mixed-method studies from high-income countries complement the clinical trials, focusing on CRP POCT adoption barriers and the attitudes and practices of participating healthcare workers (Bustinduy et al., 2017; Hardy et al., 2017; Huddy et al., 2016; Van den Bruel et al., 2016).

Despite the growing interest in the subject, we are not aware of social research studies on point-of-care biomarker testing to guide antibiotic prescriptions in LMICs. A related body of literature has explored this subject in greater depth, namely anthropological and sociological research on rapid diagnostic testing (RDT) for malaria in low- and middle-income Africa and Asia. Studies in this area pay closer attention to the social processes underlying the introduction of a new test into an established system of healthcare practices, which can lead to unforeseen implementation challenges and consequences of seemingly simple testing technologies (Beisel et al., 2016, 2; Chandler et al., 2011, 13). Themes that have been addressed in this body of work include, for example, healthcare providers’ adherence to test results (Burchett et al., 2017; Umlauf, 2017); the implementation of diagnostic tests in private pharmacies and antimalarial-selling stores (Hutchinson et al., 2015, 2017; Visser et al., 2017); the social processes and values associated with the process of testing (Chandler et al., 2012; Hutchinson et al., 2015, 2017); or the potentially adverse effect of increasing antibiotic prescription to compensate for lower antimalarial treatment (Hopkins et al., 2017). These studies share an appreciation that RDTs’ introduction, integration, and possible interference with existing social settings can yield unintended consequences for clinical practice, healthcare-seeking behaviour, and the conceptualisation of the test itself. Based on this literature, we could expect similar dynamics in CRP POCT in LMIC settings. However, no research has yet explored whether non-malarial point-of-care biomarker testing in the context of AMR is subject to similar processes. The research gap could be attributed to the lack of analytical guidance of how the introduction of a test at the patient-health system interface could be conceptualised. We respond to this conceptual challenge through our activity space framework.

### 2.2 Analytical framework

Our study considers the introduction of CRP POCT at the patient-health system interface. We define this interface as a healthcare “activity space;” that is, a social space in which patients navigate a healthcare landscape that contains numerous and diverse health system actors, not all of which patients will know or prefer. The introduction of a point-of-care biomarker test may change the behaviours of both patients and healthcare providers, but it may also fail to do so in light of existing healthcare-seeking patterns and healthcare solutions like pharmaceutical use or other diagnostic tests. We chose the activity space framing of our analysis to study the interaction of the test with (a) the healthcare workers who use it, (b) the patients whose routine care is affected by the test, and (c) the existing patient-health system linkages that might resonate or interfere with CRP POCT.

**Fig. 1:**
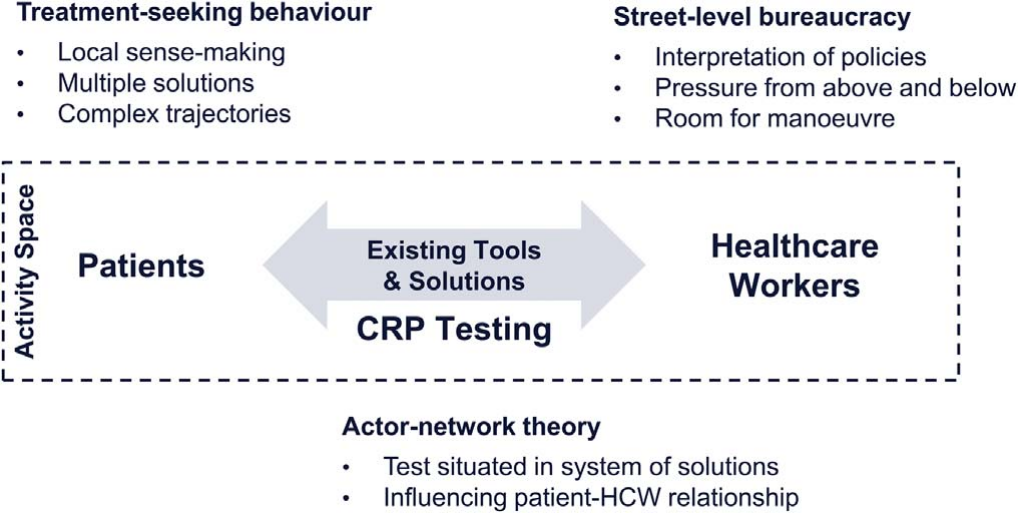
Conceptual framework for analysing role of CRP testing at health system – patient interface.

Source: Authors.

An activity space is not a theory but an analytical domain. In order to structure and guide our analysis, we borrow specific elements from different bodies of social theory, namely from treatment-seeking behaviour to explore patient behaviour in pluralistic health systems, from street-level bureaucracy to analyse the behaviour of frontline healthcare workers who face pressure from policies and guidelines as well as from their patients, and from actor-network theory to consider the role of CRP POCT in an existing network of behaviours and practices that can shape not only the impact but also the meaning of the test (summarised in Fig. 1 and described below). Drawing on these different bodies of literature does not mean that we aim at harmonising them. Rather, we follow a constructive approach that borrows from these theoretical strands and situates the CRP POCT in its social environment to understand its meaning as well as its possible (social) implications.

We adopt elements from the following three bodies of literature to structure our analysis:

1 **Treatment-seeking behaviour:** This literature emphasises, among others, the role of local sense-making processes, the multiplicity of existing healthcare solutions, and the emergence of complex and/or unforeseen healthcare pathways when a person becomes ill (Beals, 1976, 184–185; Colson, 1971, 234–236; Kroeger, 1983, 149; Lieber et al., 2006, 469). When studying the introduction of a biomarker test, we are interested in people’s existing patterns of behaviour. In a first step to understanding the role of CRP POCT, our analysis will therefore explore patients’ behaviour during illness, and the role of antibiotics as part of this behaviour.
2 **Street-level bureaucracy:** This body of work highlights the pressure that healthcare workers face from “below” (patient demands) and “above” (policies, guidelines, and superiors) when executing their work (Lipsky, 2010). It also emphasises the freedom of frontline actors vis-à-vis these pressures, leaving them to seize “room for manoeuvre” in accordance with their job description, professional guidelines, and their personal constraints and objectives (Mosse, 2004; Sheikh and Porter, 2010). A central premise for our analysis is that healthcare workers will already employ a range of tactics to manage patient and policy demands in the context of antibiotic use, not all of which will necessarily appear as “desirable” from a public health perspective. We will analyse these practices in order to understand the range of prescription behaviours and tactics that CRP POCT potentially influences.
3 **Actor-network theory:** Within the actor-network theory literature, not merely humans but also objects can be understood to have agency—that is, to influence relationships and prompt action instead of being merely subject to instrumental usage (Coupaye, 2009; Latour, 2005). We will assume that through interactions and overlaps with other healthcare practices and solutions, CRP POCT may not only compete with other options, but that it can take on new and potentially unforeseen social roles. While our analysis in this paper does not foreground CRP POCT in the same manner as an actor-network analysis would, we will start from the accepted premise that new interventions like biomarker tests are not introduced into a vacuum, but into a system of healthcare solutions with which the test potentially interacts and competes (Polgar, 1963, 411–412). Our analysis will then explore the various roles that CRP POCT fulfils in light of patients’ healthcare-seeking behaviour on the one hand, and healthcare workers’ prescription behaviour on the other hand.

In summary, in order to understand the social implications of CRP testing in Northern Thailand, we locate patients, public healthcare workers, and the intervention in an activity space framework that focuses on patients’ complex healthcare-seeking processes, healthcare workers’ prescription strategies, and the CRP biomarker tests’ potential influence in altering the existing relationships between patients and healthcare workers. For our analysis, we will first situate the antibiotic policy and its use in the local historical and social context, after which we will examine the perspectives of patients and healthcare workers and then explore how the introduction of CRP POCT alters the given assemblage of care. We expect that CRP biomarker testing can influence healthcare workers’ and patients’ behaviours beyond the instrumental provision of diagnostic information.

## 3 Materials and methods

### 3.1 Site description

This study is situated in Chiang Rai province in Northern Thailand. According to the 2015 Household Socio-Economic Survey, the North is Thailand’s poorest region with an average monthly household income of THB 18,952 (approx. GBP 450) compared to the national average of THB 26,915 (approx. GBP 650) (National Statistical Office, 2016). Chiang Rai is a decentralised province where the bulk of economic activity is generated in the northern border areas. Our field site—the capital city district Amphoe Mueang located in central Chiang Rai—comprises heterogeneous social groups ranging from Thai urban dwellers to remotely located ethnic minority populations, who commonly work in agriculture or as daily labourers and who experience physical and linguistic obstacles to accessing public healthcare (or find access to hospitals altogether impossible during the monsoon season).

In terms of its health system, Thailand has undergone rapid progress, indicated for example by an increase in female life expectancy by 9.8–78.6 years and a decrease in infant mortality from 35 to 6.4 deaths per 1000 live births between 1990 and 2013 (WHO Regional Office for South-East Asia, 2017, 9). A major development during this period was the extension of universal healthcare in 2002, with an associated expansion of primary care facilities and a reduction in out-of-pocket healthcare payments from 33% of total health expenditures in 2001 to 12% in 2014 (Jongudomsuk et al., 2015, 87; World Bank, 2017). The current structure of the health system on the provincial level comprises a provincial hospital (the Chiang Rai Prachanukroh Hospital), which oversees the District Health Office (covering 50,000 people on average) and the subordinate health centres, which cover on average 5000 people at the primary care level (Jongudomsuk et al., 2015, 23–24, 86). Primary care providers like nurse practitioners can prescribe antibiotics (Sumpradit et al., 2015, 47–48), and widespread village-level health volunteers (assigned by public health centres) as well as private-sector and informal providers accompany public healthcare provision (Chuengsatiansup and Srivanichakorn, 2007, 5; Jongudomsuk et al., 2015, 133).

### 3.2 Study design

This social study took place as part of a larger experimental trial on the introduction of CRP POCT in primary care settings in peri-urban and rural Chiang Rai district from June 2016 to August 2017 (ClinicalTrials.gov Identifier: NCT02758821). The primary objective of the trial was “to assess the impact of CRP [POCT] on healthcare workers’ antibiotic prescribing in patients presenting to primary healthcare centres with an acute fever or a recent history of fever,” thereby measuring in particular whether antibiotic prescriptions were safely reduced (“safely” e.g. in terms of symptom duration and severity of symptoms) and/or better targeted towards bacterial infections. Participating fever patients were recruited across six health centres and randomised into one of two treatment groups (with different CRP cut-off levels for antibiotic prescription) and one control group, with 200 adults and 200 children enrolled per group (i.e. 1200 in total). The patients were provided with brief information about the role of antibiotics and the risk of antibiotic resistance as part of the consent process, and posters summarising the study were displayed in the waiting areas. The treatment groups were also presented with a 5-min animated video presented by a Thai doctor, who explained antibiotics (using technical terms and the word “germ killer” [see Section 5.1] alongside antibiotic names and images), AMR (drawing on notions of bacteria and viruses), and CRP POCT (showing laboratories, scientific graphs, and bacteria and viruses) (The video is available at https://youtu.be/hJnxSwbqWOE).

The treatment groups were tested for CRP at their first visit. Following the screening of fever patients, dedicated research study nurses administered the 5-min CRP test. The process involved a finger prick to draw a drop of blood (10 s), to which would be added a dilution liquid (30 s), a conjugate (30 s), and a washing solution (20 s), after which a NycoCard II^®^ reader was used to analyse the solution, providing a quantitative CRP result within 5 min (the process is illustrated in Axis-Shield PoC AS, 2016). Patients witnessed this process and received explanations from the study staff. The study staff subsequently supplied the CRP POCT results to the consulting healthcare worker to aid their prescription decision, but healthcare workers were encouraged to base the ultimate treatment decision on their own clinical judgement. The control group had nasopharyngeal swabs and a venous blood sample taken. All participants including control also had a physical examination, reported their medical history, and provided a urine sample on enrolment. Follow-up visits for all participants involved CRP POCT together with another urine sample and follow-up questions to help gauge clinical recovery. Written consent was obtained from all participants. The research was approved by the University of Oxford Tropical Research Ethics Committee (ref. 49-15), Mahidol University Faculty of Tropical Medicine Ethics Committee (ref. 16-015), and the Chiang Rai Provincial Public Health Office Ethics Committee (ref. CR 0032.002/3516).

The experimental study design prevented us from adopting observational and/or ethnographic research methods, due to which we relied on qualitative data from semi-structured interviews and focus group discussions with fever patients and healthcare workers (the purpose of the focus groups was to triangulate and to explore the themes arising from the semi-structured interviews in greater depth). The data were collected in August 2016 and May 2017, involving pre- and post-intervention patients and healthcare workers. All 21 participating healthcare workers (including health centre principals) partook in the interviews, seven of whom were interviewed prior to the intervention. Patient sampling was purposive, geared towards maximum variation. The variables that informed the sampling strategy were the patients’ study groups (pre-intervention/control/treatment group), whether they received an antibiotic (yes/no), gender (male/female), age (guardian of a child below 18 years/18–49/50+), and education (below/above primary education). We sampled the patients from logs of fever patients maintained by the clinical trial and the health centres (for pre-intervention patients), and ultimately recruited 27 fever patients for semi-structured interviews and 10 patients for three focus group discussions (male/pre-intervention, female/post-intervention, guardian/post-intervention). We chose maximum variation sampling considering the exploratory nature of this research, for which we aimed to capture the spectrum of behaviours and conceptions among primary healthcare users, and their implications for the role of the CRP POCT. By studying the perspectives of patients and healthcare workers within and outside of the clinical trial setting, we were able to explore the role of CRP POCT at the interface of these social agents more comprehensively.

We based the interview guides (see Supplementary Material) on our analytical framework. Healthcare worker interviews covered the structure of everyday work, antibiotic prescription practices, and how CRP POCT relates to these practices. Patient interviews addressed treatment-seeking behaviour, the landscape of healthcare providers, and healthcare experiences including CRP testing. A drug card developed with the help of study staff and a local pharmacist facilitated the patient interviews (see Supplementary Material). It was employed as a tool to help recall medicines used during an illness and contained pictures of common medicines in the form of pills and capsules (rather than their packaging). While the drug card aided recall, it was less useful for deciding firmly on the type of medicine used by the respondent because many antibiotics looked alike (e.g. red-black capsules for penicillin and cloxacillin) and non-antibiotic medicine like the traditional Thai herbal medicine *fah talai jone* also appeared in similar capsules (this, however, enabled us to study for instance the theme of look-alike non-antibiotic alternatives in greater depth).

All interviews were conducted in Thai, audio recorded, and transcribed and translated together with hand-written field notes. The translation of the transcripts was meaning based (rather than literal translation) and conducted by the research team member who carried out the interviews (NC). The translation took place with daily conversations with the lead social researcher (MJH; both were present in all interviews), which helped interpret difficult concepts (e.g. related to popular concepts of antibiotics, idioms). Annotations and notes in the transcripts provided further information on the interview setting, the interviewer-respondent interaction, and the interpretation of the respondent’s statements. The resulting qualitative data comprised 51:39 h of recorded material from 58 participants. We used thematic analysis and our analytical framework formed the basis for the initial themes that guided the qualitative analysis. Emerging and sub-themes were then derived from the qualitative material. The lead researcher (MJH) coded the data in Nvivo 11 (QSR International, 2017) using the translated transcripts, and all themes were discussed with and checked for consistency and authenticity by the Thai research team member (NC). The resulting themes were subsequently discussed in the broader research group. We analysed the focus group discussions and semi-structured interviews separately, but, due to their supplementary purpose, the focus group discussions speak to and triangulate the themes from the semi-structured interviews. We will therefore present excerpts from semi-structured interviews and focus group discussions side-by-side to exemplify the themes in the “Results” section (Section 5).

## 4 Case study context

### 4.1 Recent developments and current state of antibiotic policy and use

In this section, we will situate the current state of antibiotic policy and use in Thailand in their historical and social context. Thailand has been described as a context of high antibiotic consumption, indicated for example by widespread over-the-counter antibiotic access (Apisarnthanarak et al., 2008; Khamsarn et al., 2016, 272) and estimated direct costs of drug-resistant infections of up to 1.6% of total national health expenditure (Phumart et al., 2012, 358). Thai health policy has been adapting to this problem at least since 1998 when AMR surveillance was initiated, but only recently have efforts become more integrated (Tangcharoensathien et al., 2017). Among several recent initiatives is the 2007 “Antibiotic Smart Use” campaign to promote the “rational use of antibiotics” (Sumpradit et al., 2012, 905). It targets antibiotic prescriptions and patient behaviour through activities including treatment guideline training, addressing vernacular notions of antibiotics as “anti-inflammatory medicine” (“*ยาแก้อักเสบ*” or “*yah kae ak seb*”), and promoting the prescription of herbal medicine for mild and non-bacterial infections (*andrographis paniculate*, colloquially known in Thailand as “*ฟ้าทะลายโจร*” or “*fah talai jone*”) (ReAct, 2016, 5; So and Woodhouse, 2014, 84; Sumpradit et al., 2012, 907). AMR is also visible in the Thai National Strategic Plan on Antimicrobial Resistance, which aims to reduce AMR-related morbidity by 50% and antibiotic prescriptions by 20% by 2021 (MPH and MAC, 2017). According to recent reports, current antibiotic prescription in Thailand is comparatively moderate with a mildly declining trend (WHO SEARO, 2016), contrary to many of Thailand’s regional peers. For example, a review of public-sector antibiotic prescriptions in the WHO Southeast Asia region by Holloway et al. (2017, 11) suggests that Thailand has the lowest rate of prescriptions for outpatients (12% vs. unweighted regional average of 39%) and the second-lowest rate of prescriptions for patients with upper respiratory infections (43% vs. average of 61%).

In our research setting in Chiang Rai, elements of these antibiotic policy developments had begun to diffuse into antibiotic prescription practice on the primary care level. Guidelines have become increasingly more restrictive, prescription monitoring from higher administrative levels has intensified, and antibiotic prescription has been managed more quantitatively. At the same time, local activities also emerged at the health centre level, including posters, information leaflets about antibiotic use, or monitoring of local stores to check whether they sell prescription drugs over the counter. Local antibiotic prescription and usage data from Chiang Rai is scarce. A survey of 25 villages in Chiang Rai in the mid-1990s found that all of them had antibiotics locally available, typically through informal channels and comprising various brand names, types, and mixtures with other medicines (“*ยาชุด*” or “*yah shood*,” meaning “medicine sets”) (Sringernyuang, 2000, 54, 58). Antibiotics in this survey accounted only for a small share of pharmaceutical treatment of illnesses (used in 3% of all recorded illnesses) compared to analgesics and cough relievers, but the widespread availability suggests at least a general acquaintance with antibiotics among the study population (Sringernyuang, 2000, 90). At the same time, the emphasis on *yah shood* in this and other studies (e.g. Chuengsatiansup et al., 2000, 19) is at odds with our observations, as we witnessed only isolated second-hand reports of *yah shood* use during our one-year research period in Chiang Rai (contrary e.g. to related research that we carried out in Yangon; Khine Zaw et al., in press).

### 4.2 Historical roots and the social meaning of Thai antibiotic use

Historical context helps to understand the roots of widespread medicine use (including antibiotics) and the implications of the current AMR policies. Over the past century, approaches to health and medicine in Thailand gradually evolved from an elite- and modernism-driven to a more participatory and multi-disciplinary discourse from the 1970s onwards (Muksong & Chuengsatiansup, forthcoming). During the modernist paradigm (which overlapped for instance with the first Five-year National Health Development Plan in 1962; Sringernyuang, 2000, 29), health in Thailand became “wedged to [the] biomedical model of health” and entailed the “medicalisation of health” (Muksong & Chuengsatiansup, forthcoming, 10). The associated primacy of Western pharmaceuticals in the Thai health system during the modernisation discourse was followed by a primary healthcare approach and the promotion of traditional Thai medicines in the 1980s, which are now used and promoted as an alternative to pharmaceutical treatment in general and antibiotic prescription in particular (Chuengsatiansup and Srivanichakorn, 2007, 3–4; le Grand et al., 1993, 1025; So and Woodhouse, 2014, 84; Tantipidoke, 2013, 4–5; WHO SEARO, 2016, 28–29). However, being a multidisciplinary and contested field, a plurality of actors has been driving health sector development in recent years, involving not only community-based organisations but also for example private for-profit healthcare providers that had grown rapidly as a means to fill gaps in patchy rural health services (and public finances); yet private providers also soon developed strong interest groups and acquired a reputation for special if not superior medical services like injections (Bennett and Tangcharoensathien, 1994; Chuengsatiansup and Srivanichakorn, 2007, 7; Sringernyuang, 2000, 69). In addition, the pluralistic landscape of actors is uneven, as the Thai health sector continues to be dominated by the biomedical logic of medicine, which also penetrates the practice of traditional Thai medicine with an increasing degree of medicalisation and commercialisation (Tantipidoke, 2013, 4–5).

Because of these health sector developments, Western medicine has offered a widening portfolio of treatment even in rural contexts while the popular knowledge about traditional Thai medicine has broken down (Sringernyuang, 2000, 33; WHO SEARO, 2016, 28). During this process, the sources of treatment and medicine have gradually become more formalised, shifting—albeit incompletely—from traditional healers, local stores, and itinerant sellers to public and private clinics and hospitals as well as registered pharmacies (le Grand et al., 1993, 1030; Sringernyuang, 2000, 35). These developments are not entirely unproblematic because informal stores and traditional healers do not only fulfil functions of bridging healthcare gaps, but also provide alternative systems of medicine and socially accessible healthcare when for instance minority groups face discrimination or linguistic barriers in the public healthcare sector (Apidechkul et al., 2016, 69; le Grand et al., 1993, 1027; Tantipidoke, 2013).

As described above, widely accessible antibiotics even in rural settings suggest that they are an integral part of Western medicine in Thailand. At the same time, the uses of antibiotics do not seem to be universal but concentrated around specific illnesses and symptoms. For example, Sringernyuang (2000, 117–135) describes a case study of a form of pelvic inflammatory disease that is popularly understood as uterus inflammation (“*มดลูกอักเสบ*” or “*mot look ak seb*”). The author hypothesises that pharmaceutical advertising had initially created a conceptual link between this form of “inflammation” and specific antibiotic brands, which later evolved into the generic use of antibiotics as “anti-inflammatory drugs” through experimentation (e.g. because patients could not afford the original brand). The establishment of this conceptual link was facilitated by the mismatch between the biomedical logic of antibiotics and their popular notion as “anti-inflammatory medicine” (considering the absence of notions of infection, bacteria, and viruses in Thai traditional medicine), and people’s uncertainty resulting from ambiguous definitions of the disease (Sringernyuang, 2000, 133–135). This in itself does not imply popular recognition as a “wonder drug” as is occasionally claimed the clinical and social sciences literature (e.g. Zellweger et al., 2017, 2969). Rather, antibiotics appear to fulfil two purposes. On the one hand, the persistent link between “inflammation” and antibiotics as “anti-inflammatory medicine” (Khamsarn et al., 2016, 271) helps patients to establish a “popular explanatory model” (i.e. mental framework) of illness and cure, thereby reducing uncertainty in an ambiguous experience that merges biomedical and traditional notions of medicine (Sringernyuang, 2000, 133–135).

On the one hand, antibiotics satisfy generic expectations for medicinal treatment alongside other pharmaceuticals like analgesics (le Grand et al., 1993, 1031). This expectation is rooted in the gradual medicalisation and formalisation of healthcare in Thailand and corresponds (a) to a “cultural context where people usually perceive that no treatment is taking place unless drugs are prescribed” (Sringernyuang, 2000, 37), (b) to a higher perceived effectiveness of Western and expensive as opposed to traditional Thai medicine, (c) to an environment where 19 out of 20 illness episodes are being treated with medicine, and (d) to the now widespread self-treatment with medicine especially among poorer and socially marginalised parts of the Thai population (Chuengsatiansup et al., 2000, 8–9, 28–29; Sringernyuang, 2000, 37, 80). The seemingly declining rate of antibiotic prescription thereby masks high levels of general medicine use, indicated for example in a reported average of 2.8 medicines prescribed per patient in public primary care settings (WHO SEARO, 2016, 47). The policy attention on reducing antibiotic prescription therefore raises questions about the demand for other medicines like analgesics or non-steroidal anti-inflammatory drugs, which have their own meaning and side-effects, which were commonly available in rural Chiang Rai at least until recently, and which may not receive the same degree of global and local policy attention as antimicrobials (Sringernyuang, 2000, 45–50).

In summary, CRP POCT is introduced into a context where—albeit still worryingly high from a global health perspective—antibiotic use is low compared to other countries in the region, following extensive policy action and local initiatives. The historical pattern of antibiotic use has to be considered alongside other Western pharmaceuticals, which people have come to expect as the basis for medical treatment. The review thereby highlights that seemingly “individual or irrational behaviour” around antibiotic use is structured by broad social and historical processes in which pharmaceuticals have been heavily marketed and citizens have been encouraged to think that care can only be enacted if pharmaceuticals are provided (we thank an anonymous reviewer for these reflections). Among the many medicines that people access for common ailments, antibiotics represent a comparatively small part and may therefore not be considered the stereotypical “wonder drug.” Yet, for specific symptoms and conditions, they have integrated consistently into a local system of medicine and help reduce uncertainty among patients. Potential reductions of antibiotic prescriptions through CRP POCT could therefore relate to generic functions of antibiotics as good care (alongside other medicine), and to specific functions of reducing uncertainty for conditions that are conceptually linked to antibiotic use.

## 5 Results

### 5.1 Patients’ healthcare-seeking behaviour

To understand the role of the CRP test at the patient-health system interface, we first document the conceptions and behaviours around antibiotic use in Chiang Rai. We focus on the notions of “antibiotics” encountered in the field, and the various treatment-seeking patterns that emanate partly from the local conceptions.

Varied local conceptions around the concept of antibiotics (summarised in Table 1) emerged as a central theme in people’s treatment-seeking behaviour. The Thai term for “antibiotic” was rarely uttered among respondents and our interviewees rather referred to “anti-inflammatory medicine,” “microbe [or germ] killer” and “sore throat medication.” Members of different ethnic groups like Akha or Lahu reported that their language would not have an equivalent term for “antibiotic” or the Thai “*yah kae ak seb*;” local equivalents of “antibiotic” would rather refer to medicine that “*relieves the pain*” – and use it for muscle pain accordingly. Such was the case in an interview with an Akha respondent (excerpt from interview notes):

*Their language Akha does not have a word for “anti-inflammatory drugs” (like Lahu). It refers to “getting rid of the pain.” They learned the link between muscle pain and anti-inflammatory drugs [i.e. antibiotics] through their experiences at the health centre. Their sole sources of health information are the health centre and the hospital*. (interview notes, male guardian, no formal education, children in both treatment and control groups)

**Table 1:** Local expressions for “antibiotics” encountered in Chiang Rai.

English	Thai	Explanation
“*Antibiotic*”	*ยาปฏิชีวนะ* (“*yah pa ti chee wa na*”)	Technical term with Pali roots, rarely used (e.g. higher education levels); linked to varied modes of use, e.g. sole dependence on doctors’ advice as well as self-medication for sore throat
“*Anti-inflammatory drug*”	*ยาแก้อักเสบ* (“*yah kae ak seb*”)	Common vernacular expression of antibiotics; sometimes referring to anti-inflammatory drugs; often linked to sore throat, muscle pain, wounds, acne
“*Microbe/germ killer*”	*ยาฆ่าเชื้อ* (“*yah kah chuea*”)	Vernacular description of antibiotics; may also include e.g. stomach medicine or rubbing alcohol; linked to wide range of illnesses including fever in some instances
“*Sore throat medication*”	*ยาแก้เจ็บคอ* (“*yah kae jeb koh*”)	Vernacular description linked to sore throat as commonly treated symptom; can also refer to cough medicine/drops
“*Amoxicillin*”	*แอมม็อกซี่* (“*amoxy*”)	Vernacular expression of antibiotics as uttered literally, specific reference to antibiotics but relatively uncommon (e.g. higher education, healthcare workers); uses similar to *yah pa ti chee wa na*
“*Medicine that relieves the pain*”	[no local language equivalent of Thai “antibiotic” or “anti-inflammatory drug”]	Description of antibiotics without local language equivalent (e.g. Akha, Lahu); linked esp. to use for muscle pain

Note. The order in which these concepts are presented does not imply a hierarchy of the terms, behaviours, or groups.

Our interviews thereby consistently pointed at connections between vernacular concepts of antibiotics (variously defined) and their use for specific symptoms, similar to their link to “*มดลูกอักเสบ*” (“*mot look ak seb*”) in Sringernyuang (2000, 132–135) twenty years ago. In our case, it appeared that antibiotics as “anti-inflammatory drugs” resonated with people’s reported conceptions of sore throats (rather than fever) and muscle pains as forms of bodily inflammation. Only few of the interviewed patients would consider treating a simple fever with antibiotics and would rather depend on healthcare providers to prescribe them. Rest, sponge baths, and paracetamol appeared to be the common treatment for a fever instead. Furthermore, notions of antibiotics as “sore throat medication” and “pain reliever” were uncommon but directly linked to the use for specific symptoms, which accompany fevers only occasionally. (While our sample of fever patients in public primary care settings does not allow a more comprehensive mapping of the link between antibiotic conceptions and treatment-seeking patterns, we are currently collecting survey data to test this relationship on the provincial level.)

Antibiotic access had further drivers beyond language. For example, logistical access constraints (e.g. landslides during the rainy season) would lead people in mountain villages to rely on local informal and private healthcare providers when otherwise they would be drawn to free healthcare services and medicine from public facilities. Another important theme was that patients would ascribe different values to medicine received from various sources, with public and private healthcare providers offering better-quality medicine than local stores – possibly stemming from the modernisation, formalisation, and medicalisation trends in the Thai health system over past decades. As a result, sets of mixed medicines called “*ยาชุด*” (“*yah shood*”) – though widely known – appeared to be used rarely. Likewise, antibiotic access through local stores and private pharmacies was primarily a resort for patients who would require help outside of the opening hours of the public health centres, who would emphasise their lack of time, or who would value their own health beneath that of family members. An example of the values associated with medicine from different healthcare providers is the following exchange with a male guardian, whose daughter would receive antibiotics at a health centre, who would buy antibiotics for himself at a pharmacy (requesting “*anti-inflammatory*”), and who would buy antibiotics for his chickens from a local store:

R: *“It’s one baht a pill* [approx. GBP 0.02]*, normally the red and black* [presumably cloxacillin based on drug card] *you can get them from the shops for one baht a pill.”*

Q: *“Do you buy those? Do you buy the red and black to use?”*

R: *“I buy it for the chickens.”*

Q: *“But you don’t use it.”*

R: *“I don’t use chickens’ medicines.”* (male guardian, primary education, treatment group)

His case illustrates the treatment-seeking patterns that result from the ascribed value of medicine from different healthcare providers. More generally, guardians would maintain that their children’s health is valuable and that they would not want to risk complications by buying medicine from informal stores. In contrast, adults themselves argued that they know their body, that they have more experience with illness and medicine compared to their children, and that they can tolerate stronger and/or lower-quality over-the-counter medicine. These differences in ascribed effectiveness of medicine from different sources are visible in statements such as,

*“the health centre’s medicines work with my son, but not with the grandparents,”* (female guardian, secondary education, pre-intervention group), and

“[I do not buy medicines for my children] *because the kids have low immunity and they cannot take strong medicines* [from drug stores]*. Us adults can take medicines in* [the form of] *pills, right?*” (female guardian, primary education, intervention group),

And were confirmed in the focus group discussion with guardians as well:

*“If it’s my child, I’d have to take her to the doctor. But if it’ me, I can just buy the medication and take it”* (focus group response of female guardian, secondary education, treatment group).

Public healthcare providers were therefore often the preferred point of treatment for the children. Adults would rather use medicine they had stored at home or simply buy antibiotics at pharmacies and local shops that are more “*convenient*” to access.

Like patients who seek medicine through private or informal channels, those who actually visit public healthcare facilities would commonly expect to receive medicine to make their visit worthwhile. The resemblance to historically grown expectations of medicine as “good care” is evident in statements like,

“*I’m here* [in the health centre] *in hope to receive medicines, you know?*” (woman, primary education, treatment group), and

R: *“Isn’t that right* [to expect medicine]*? When I go to a doctor … I would expect from the doctor at the health centre to receive medicines to relieve* [my symptoms]*. Because if I don’t receive anything, I would have to find other ways* [to cure myself] *because I’m sick, you know? I would have to find a way.”*

Q: *“Hm. Has it ever happened that you went and didn’t receive medicines to take home?”*

R: *“It happened in the past, but … even when I went to the hospital. You know what I got? Six paracetamols from* [waiting] *the whole day.”* (man, no formal education, treatment group)

These general expectations for pharmaceuticals in the case of fever may neither be limited nor linked to antibiotics as “anti-inflammatory medicine” or “sore throat medication” unless associated symptoms of “inflammation” are present. The conversations rather suggested that patients expecting an antibiotic could accept other treatment or advice on how else to remedy the illness, provided that the substitute treatment resonates with their generic expectations of good care.

In summary, treatment seeking in our sample of Chiang Rai fever patients more commonly involved informal and private providers for people who faced access constraints to public healthcare facilities and for adults in general. The type of illness thereby influenced the healthcare provider choice not so much directly, but indirectly through considerations of what types of medicine would be suitable to treat the illness, and where they could be obtained at an acceptable level of quality. The conceptual links between antibiotics and treatment were varied, and only some included the antibiotic treatment of fever. At the same time, antibiotics also appeared to fall under the more general category of pharmaceuticals that represent “good care.”

### 5.2 Healthcare workers’ prescription behaviour

When prescribing antibiotics (commonly reported e.g. for sore throats, pneumonia, infected wounds, tonsillitis, diarrhoea, or urinary tract infections), nurses negotiate constraints from “above” (policy and administration) and from “below” (patients). From “above,” diagnosis and prescription follow clinical practice guidelines, on which most interviewed nurses were updated annually through conferences. These conferences also introduced new policies and initiatives relating to AMR, which was evident by occasional references to the “Antibiotic Smart Use” campaign. Influences on prescription practice emerged also from the provincial hospital, which had been managing and monitoring the antibiotic supply to the health centres more closely. Healthcare workers thereby suggested that more restrictive supply management could help reduce antibiotic over-prescription because health centres “*wouldn’t have medicines to prescribe for the patients* […]*, so that’s the end, problem solved*” (principal, pre-intervention site).

From “below,” healthcare workers occasionally faced unspoken expectations or explicit demands for antibiotics. For example, an intervention-site nurse described how she would normally adhere to guidelines when a patient presents with sore throat, but antibiotic non-prescription might also lead some patients to request antibiotics: “*If I don’t prescribe* [an antibiotic] *to these, I might get a dirty look. They know, the villagers, ‘Oh do I not get the anti-inflammatory? Do I not get the germ killer?’*” (nurse, intervention site). Nurses reported that between one patient per year to 50% of all their outpatients would articulate such demands, but it was commonly raised as a theme that interfered with nurses’ adherence to routine prescription guidelines (esp. if they lived within the local community).

**Fig. 2:**
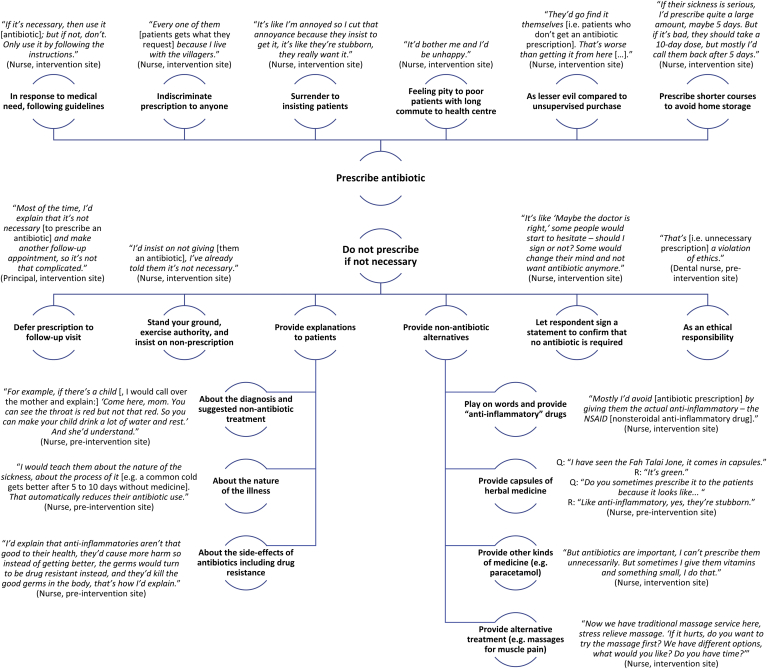
Tactics and justifications relating to antibiotic non-/prescription among Chiang Rai nurses, including example statements.

Source: Authors, based on qualitative analysis.

A portfolio of practices and tactics involving prescription and non-prescription of antibiotics resulted from nurses’ negotiations of their job description, clinical guidelines, the broader policy environment, and patients’ explicit and implicit demands. These practices are summarised in Fig. 2 together with example quotes. Most prescription decisions were not deemed problematic, but when faced with patient demands, nurses may prescribe antibiotics as they (a) surrender to continuously insisting patients, (b) pity poor patients who incurred travel costs, (c) consider it the lesser evil to provide supervised antibiotic prescriptions than having the patient buy medicine from informal stores, or (d) prescribe shorter courses to prevent patients from storing leftovers at home. Nurses also reported tactics to dissuade patients from unnecessary antibiotic use. While some “stood their ground” or tried to convince patients through additional explanation, another common tactic was to provide other medicine (e.g. paracetamol) or treatment (e.g. massages) as alternatives to antibiotics. Nurses thereby also provided patients with the herbal medicine *fah talai jone* as a treatment for sore throat (also because its capsules resemble antibiotics), and they tapped into conceptual ambiguity to prescribe an “anti-inflammatory drug” (e.g. nonsteroidal anti-inflammatory drugs) as literally requested by patients expecting an antibiotic. Other practices involved having the patient sign a statement that they understand the lacking necessity of an antibiotic to deter them by reflection, or the deferral of the prescription to (unlikely) follow-up visits if the patients’ symptoms would not improve.

The principal insight from this analysis is that healthcare workers decide actively about antibiotic prescriptions (rather than being passive prescribers), and that they employ a range of solutions to limit over-prescription. That healthcare workers do not constitute an “empty vessel” for the biomarker test introduction might not be surprising in itself, but the behaviours outlined in this section represent a set of tactics and solutions that enable healthcare workers to make ethically and socially laden prescription decisions under partial uncertainty. The CRP POCT enters and interacts with these practices, as we will highlight below and discuss further in Section 6.

### 5.3 Roles of C-reactive protein point-of-care testing

The reception of the test was positive among the interviewed patients and healthcare workers. Nurses noted the additional information that aided (though not replaced) the existing diagnostic process, and its results are “*better than only using words*” (nurse, intervention site) when trying to convince patients. Concerns arose only speculatively about the workload and resource implications of a possible future scale-up of CRP testing. Patients, too, responded positively to the CRP test. Aside from passing worries about the pain of the finger prick and initial uncertainty while they waited for the test results, the respondents expressed a liking for the testing procedures they underwent, suggesting for example that the treatment is “*better with the blood test. They take care of you*” (female guardian, secondary education, treatment group). The overall optimistic responses suggest that CRP POCT supports nurses and satisfies patients, but the effect on antibiotic prescription practices is more nuanced for both parties.

On the side of the healthcare workers, the role of the test is manifest for instance in its provision of additional information “*that helps us evaluate the patients faster*” (nurse, intervention site), which can hide nurses’ clinical uncertainty. One nurse (who was enthusiastic about the clinical trial) explained that CRP POCT results might disagree with his own judgement. Asked whether he trusts his own judgement in this case, he likened CRP POCT to laboratory testing, suggesting that “*I’ve got to trust the lab result* [laughed]. […] *If the result says so, that means there’s got to be something wrong somewhere in the body*” (nurse, intervention site). Confidence in a CRP-aided prescription process can therefore arise from confirming an existing diagnosis but also from the test’s ability to circumvent diagnostic uncertainty in guiding treatment. Yet, the influence of CRP POCT was not pervasive, as other nurses relied on their own judgement to override test results (as the clinical study encouraged them to), and they continued referring uncertain and risky cases to the hospital. If professional judgement and CRP POCT results agreed that an antibiotic was not necessary for treatment, then the test influenced the patient-nurse interaction by helping to “convince” the patient of the credibility of the nurses’ recommendations: A nurse considered that patients understand the test as “*scientific evidence. It can assure the patients explicitly.* […] *In some cases, I would say, ‘The lab result looks like this. There’s no need* [for an antibiotic]*.’*” (nurse, intervention site).

On the patient side, too, the test goes beyond being a mere diagnostic tool to detect bacterial infections. Contrary to its designed purpose and despite an information video provided to participants during the clinical trial, patients consistently re-interpreted the CRP finger prick test as a comprehensive blood test, able to detect any disease in the human body, or, for instance, “*to know what’s wrong with my daughter because they* [i.e. the doctors] *are afraid of her having a lethal disease*” (male guardian, primary education, treatment group). While this indicates that the test is valued for its ability to rule out uncertainty about the patients’ illness, the overall positive perception of (and reported trust in) CRP POCT is potentially undermined by patients’ demand for information about alternatives to antibiotic treatment, continued expectations to receive medicine that compensates for tedious journeys to the health centre, and access to alternative treatment options if their symptoms do not improve despite CRP-POCT-guided advice (e.g., “*If it doesn’t get better, I wouldn’t let them do the prick* [again]; ” woman, primary education, control group). This suggests that the care experienced by the CRP POCT is only a partial substitute for the expectations of “good care” as pharmaceutical treatment.

In summary, CRP POCT does not only yield diagnostic information. The test emerges as a new element in the assemblage of care, in which it fulfils important social functions of “good care” and of reducing the ambiguity that both patients and healthcare workers experience during their encounter – similar though not identical to antibiotics. We will discuss and interpret these findings in the social and historical context of antibiotic use in the next section, together with their potential implications for healthcare and antibiotic provision in Thailand.

## 6 Discussion

### 6.1 Situating CRP POCT within an existing network of solutions

This paper set out to study the role of CRP POCT on primary-care-level antibiotic prescription in Chiang Rai. Our qualitative analysis suggested that the introduction of the test into an existing system of practices at the patient-health system interface leads to a redefinition of its role from a technical to a social device. In this section, we discuss the findings from our analysis in light of the social and historical context and the limitations of our study.

Antibiotic use in Chiang Rai corresponds historically to generic expectations of Western medicine as “good care” while also relieving ambiguity among patients and primary healthcare providers (Sringernyuang, 2000, 133–135). Similarly, CRP POCT as a primary-care-level intervention for fever patients helped alleviate therapeutic ambiguity not only by reassuring patients about the nurses’ treatment decisions, but also about their health. Drawing on Coupaye (2009, 444), we could argue that this reassurance arises from the values associated with overlapping tasks: CRP POCT resembles activities that patients mainly associate with blood testing in hospital settings. Blood and its transfer are imbued with meaning (Johnson et al., 2013), and blood in our case has a particular importance as bearer of vital information about one’s health. Transferring blood to a medical expert (i.e. a nurse) for scientific analysis through a technical device like the CRP POCT bestows onto the test the diagnostic power of a comprehensive blood test. According to this explanation, patient reassurance emanated from the values that had become associated with and embedded into the POCT process, like care, reassurance, modernism, and the value of scientific evidence. This reassurance led a wide range of our respondents to trust that a low CRP level indicated good health (in simple terms, a low CRP level indicates the absence of a bacterial infection, even if a viral infection is present; and the level might also be depressed by prior antibiotic use). We hypothesise that this response is particularly pronounced if there is a mismatch between the internal biomedical logic of the CRP POCT and patients’ own “explanatory models” – which at least until recently did not commonly incorporate notions of bacterial infection (Sringernyuang, 2000, 133–135).

We also presented evidence that patients perceive the CRP POCT treatment as “good care.” At least in the context of this clinical trial, participation meant additional care from study staff who operated and explained the test in addition to extensive trial-related procedures and sample collection (e.g. urine tests), which was likely to accentuate the impression of “good care” received by the patient. Further research will have to explore the use of CRP POCT in routine care settings, and the implications it has not only on antibiotic prescription but also on healthcare-seeking patterns, social interactions between patients and healthcare providers, and the use of alternative pharmaceutical and non-pharmaceutical treatments. However, it is plausible that CRP POCT fills a gap in a setting where antibiotic prescription as another route of demonstrating and enacting care is being reduced more generally and by the CRP POCT itself. CRP POCT thereby integrates into and partly substitutes for pharmaceutical use.

At the same time, the range of patients’ healthcare-seeking patterns in the pluralistic health system of Chiang Rai means that

(a) patients link the diverse notions of antibiotics to different symptoms and conditions ranging from bacterial infections via inflammation to sore throats and muscle pain (in line with their functional descriptions), and that
(b) medicine access patterns often continue to involve (i) private practitioners that are often seen as providing special medical services compared public providers and (ii) pharmacies and local stores if patients face logistical, economic, or social obstacles to public healthcare access.

Healthcare-seeking behaviours could thus become incompatible with fever-focused CRP testing at primary care units. Such incompatibility would arise if (a) patients sought antibiotics for non-fever symptoms like acne or muscle pain, and if (b) they did not normally approach health centres for healthcare and medicine, but rather private and informal providers like pharmacies or grocery stores. The social role of the CRP POCT would then pertain only to (and replace social functions of reduced antibiotic use for) a specific sub-population of antibiotic users.

The role of the test was further defined in relation to the policy environment. CRP POCT was introduced into a setting where healthcare workers noted the policy focus on antibiotic over-prescriptions and the increasingly monitored supply of antibiotics from the Chiang Rai hospital. As nurses’ routine practice has become infused with these constraints “from above,” the role of CRP testing expanded from diagnostic support to allocative decision making that responds to an increasingly restrictive policy environment. Some respondents did indeed suggest that standardised indicators from CRP POCT could be a solution to antibiotic over-prescription. This position is reminiscent of middle-upper arm circumference bands, which according to Scott-Smith (2013, 923) introduced standardised indicators into the allocation process for nutritional support within refugee camps (we adapted his quote to our setting):

“[Measurement … allows] the [medical practitioner] to sort the population efficiently into those who will and will not receive [antibiotics], offering a regularised mechanism for [prescription], and transferring difficult ethical decisions out of the [healthcare] worker’s hands.” (Scott-Smith, 2013, 923)

As shown in Fig. 2 (which summarised the range of nurses’ antibiotic prescription tactics), the prescription process has an ethical dimension in which some nurses justified non-prescription with an ethical responsibility to abide by their clinical guidelines, while other nurses linked the prescription of antibiotics to their social responsibility towards poorer patients living in remote locations. CRP POCT enters this ethical space. As an allocative tool similar to middle-upper arm circumference bands, it can legitimise forms of antibiotic non-prescription that were previously deemed in violation with the personal ethics of the nurses. The introduction of the CRP POCT may further reconfigure the social space between patients and healthcare providers thus.

A central limitation of these interpretations is the restriction of our study sample to (a) febrile patients who accessed public health centres, and to (b) healthcare workers who participated (or agreed to participate) in the CRP biomarker test trial. We can therefore only capture treatment-seeking patterns that took place outside the public sector through circumstantial evidence of people’s experiences in previous illness episodes. Our position as outsiders was a further complication, potentially influencing responses through:

(a) power relationships indicated for example by education differentials (partial remedy: we introduced ourselves as students of the respondents to learn from them)
(b) language and cultural distance (partial remedy: we interviewed in northern Thai dialect and third-party translators)
(c) organisational constraints and insider-outsider power dynamics with healthcare staff.

It is possible that our themes thus omitted more critical and negative responses towards the health system and CRP POCT. Although our broad range of participants yielded enthusiastic as well as critical responses, we cannot ascertain the extent to which the themes are representative for the broader population of patients and healthcare workers. The clinical trial further influenced the perception of care by the patient because CRP POCT was carried out under controlled conditions by dedicated staff and alongside other tests as part of the clinical study. Methodologically, we were also limited by the absence of participant observation methods to understand the direct encounter between patients and nurses and the role of the CRP POCT therein. Although desirable, participant observation was unattainable within the given clinical trial design into which this social research study was embedded. Ethnographic fieldwork would also have enabled a deeper understanding of the CRP POCT from the perspective of actor-network theory. The conclusions of this paper should therefore be read as one among the first steps towards understanding the social nature of point-of-care biomarker testing and as hypotheses for further anthropological and sociological enquiry to study biomarker tests in the context of AMR.

### 6.2 Potential consequences of CRP POCT

**Fig. 3:**
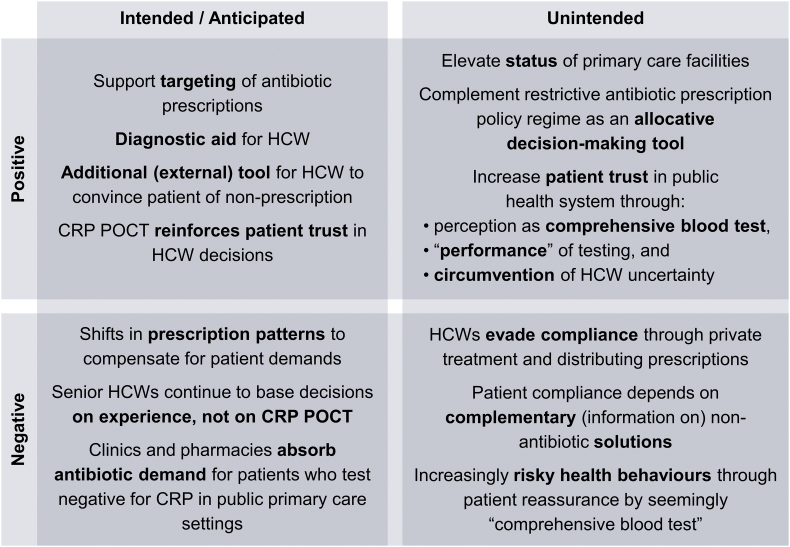
Matrix of hypothesised consequences of introducing CRP POCT.

Source: Authors, based on qualitative analysis.

Based on our analysis, we consider the possible intended/unintended and positive/negative operational consequences of biomarker testing for primary-care-level antibiotic prescriptions in Chiang Rai (summarised in Fig. 3). Considering our study limitations, these outcomes are hypothetical and require further research to ascertain their extent and impact.

The lead researchers of the CRP POCT trial expected that the test would be complementary to antibiotic policy in Thailand, reducing antibiotic prescriptions for febrile patients attending primary care, and helping healthcare workers both as a diagnostic aide and as a compliance tool for patients (whose trust in healthcare workers was expected to increase). The study team also expected that a CRP-POCT-guided decision to not prescribe antibiotics might shift prescription patterns within the participating health centres. In addition, healthcare workers were expected to comply only partially with the test (based on instructions to also rely on their own clinical judgement), and that negative antibiotic prescription decisions might lead some patients to explore alternative (private or informal) channels of antibiotic access.

While we report the quantitative results of the clinical trial on antibiotics prescriptions elsewhere, our analysis suggests that CRP POCT can realise its positive intentions and support diagnostic processes of HCWs for low-risk “*borderline*” cases. Severe cases continue to be referred to the hospital and seasoned nurses may tend to override disagreeing test results (justifiably so if the test is considered a complement to clinical practice). The test results can further help reduce over-prescription by building trust and convincing demanding patients. However, improved antibiotic targeting can entail shifts in prescription patterns to compensate for patients’ general demand for medicine, the outcomes of which cannot be ascertained without further study. Simultaneously, potentially persistent demand for antibiotics could enable private and informal healthcare providers to absorb negative CRP cases. The test may also have further unintended consequences. For example, it might increase patient trust in public health care by hiding rather than resolving HCW uncertainty, and by patients overestimating the diagnostic capabilities of the test. (We refrain here from debating whether patients *should* understand the nature of the medical procedures they undergo, focusing merely on the resulting behaviour.) Positive compliance effects may also be offset by potentially risky health behaviours emanating from false reassurances by a “comprehensive blood test” for patients with non-bacterial infections.

These outcomes are unlikely to be distributed evenly. In our study, children appeared more likely to realise the intended positive consequences because they tended to access public healthcare and their guardians complied with nurses’ recommendations. Adults rather sought help elsewhere, and, if they visited a health centre, often challenged the treatment decisions. With the clinical trial focus on fever, CRP POCT may also miss patients who use antibiotics for conditions like acne and muscle pain. Based on our analysis, we therefore hypothesise a reduction in unnecessary antibiotic prescriptions, but also a changing patient-health system relationship with adverse equity implications across generations and socioeconomic strata.

### 6.3 Relation to the literature

On the surface, our findings correspond to the qualitative clinical literature that highlighted positive patient and healthcare worker attitudes associated with the introduction of CRP POCT in high-income countries (e.g. Hardy et al., 2017; Huddy et al., 2016; Van den Bruel et al., 2016). Our activity space approach helps to put these positive attitudes into perspective. For example, the portfolio of nurses’ non-/prescription tactics confirms that CRP POCT is not introduced into an “empty vessel” (Polgar, 1963, 411–412) and highlights the interactions between the test introduction and the network of practices and its underlying determinants (e.g. a demand to compensate costly and time-consuming health facility visits rather than for antibiotics in particular; Chandler et al., 2012, 1532; Umlauf, 2017, 8–9). These interactions could entail unforeseen prescription patterns if antibiotics are not justified according to the CRP test but healthcare workers feel compelled to satisfy a patient nonetheless. The evolving prescription patterns following the introduction of point-of-care tests should be the subject of further study.

Our patient interviews reflect further themes from the medical anthropology literature, for instance values associated with testing procedures. Patient reassurance arises from a conception that the analysis of one’s blood yields treatment decisions based on full information. This argument complements themes in Chandler et al. (2012, 1531–1532), where RDTs also provided “psychological treatment” of patients, and it relates to the importance of “testing blood” in Hutchinson et al. (2015, 56). In addition, considering the varied local conceptions of illness and medicine, further research should explore how linguistic differences between implementation contexts may affect CRP POCT.

Lastly, through the activity space framework, we contribute to the understanding of point-of-care tests as technologies that co-evolve with their local context (Beisel et al., 2016, 2). This co-evolution is evident in their re-interpretation as a comprehensive blood test and their role in altering the behaviours and practices of patients and healthcare workers. Our focus on the patient-health system interface offers a novel framework inspired by healthcare-seeking behaviour, street-level bureaucracy, and actor-network theory to study the role of these and other health technologies in shaping healthcare practices and behaviours.

## 7 Conclusion

This paper studied the role of CRP POCT in Chiang Rai to reduce unnecessary antibiotic prescriptions at the primary care level. We applied a multidisciplinary analytical framework that conceptualises the patient-health system interface as an “activity space” populated with a network of practices and solutions, within which CRP POCT is introduced. This conceptualisation permitted us to draw on different theoretical threads to consider the behaviour of patients (treatment seeking), healthcare workers (street-level bureaucracy), and the test itself (actor-network theory). This work speaks to the empirical literature on CRP biomarker testing, and to the anthropological and sociological literature on rapid diagnostic testing in LMICs.

Based on qualitative analysis of responses from 58 patients and healthcare workers, we found that the test was reconfigured as a comprehensive blood test for patients and as a negotiating tool that provides a screen of certainty for healthcare workers. CRP POCT interacted with and altered existing practices that link patients and the health system, thus reinforcing healthcare worker confidence, increasing the social status of primary care facilities, and building patient trust that potentially entails risky health behaviours. The test also complements the increasingly restrictive antibiotic policy environment in Chiang Rai. However, CRP POCT focusing on fever and respiratory illnesses in public primary care settings may miss a wide spectrum of other potentially unnecessary antibiotic prescriptions, and it may lack efficacy where no dependable alternative treatment or complementary information is provided to patients.

The qualitative results are only indicative at this stage, but they suggest that CRP POCT lives up to its aspirations of improving the targeting of antibiotic prescriptions in Chiang Rai, although partly for unintended reasons. We are still at an early stage of understanding point-of-care testing and its impact on antibiotic prescriptions and healthcare behaviour in LMICs. More research is needed to ascertain effects on prescription behaviour, distributional implications on different groups of patients, and how the policy environment and healthcare practices, local perceptions of illness and medicine, and a broader set of contextual and structural factors influence the nature, effectiveness, and usefulness of point-of-care testing.
