# Supplementary material for: The social role of C-reactive protein point-of-care testing to guide antibiotic prescription in Northern Thailand
Source: Soc Sci Med. 2018 Apr;202:1–12. doi: 10.1016/j.socscimed.2018.02.018 (PMC5910303; doi:10.1016/j.socscimed.2018.02.018)
Supplement: Supplementary material [file mmc1.docx]

# The Social Role of C-Reactive Protein Point-of-Care Testing to Guide Antibiotic Prescription in Northern Thailand

# Supplementary Material

# Drug Card


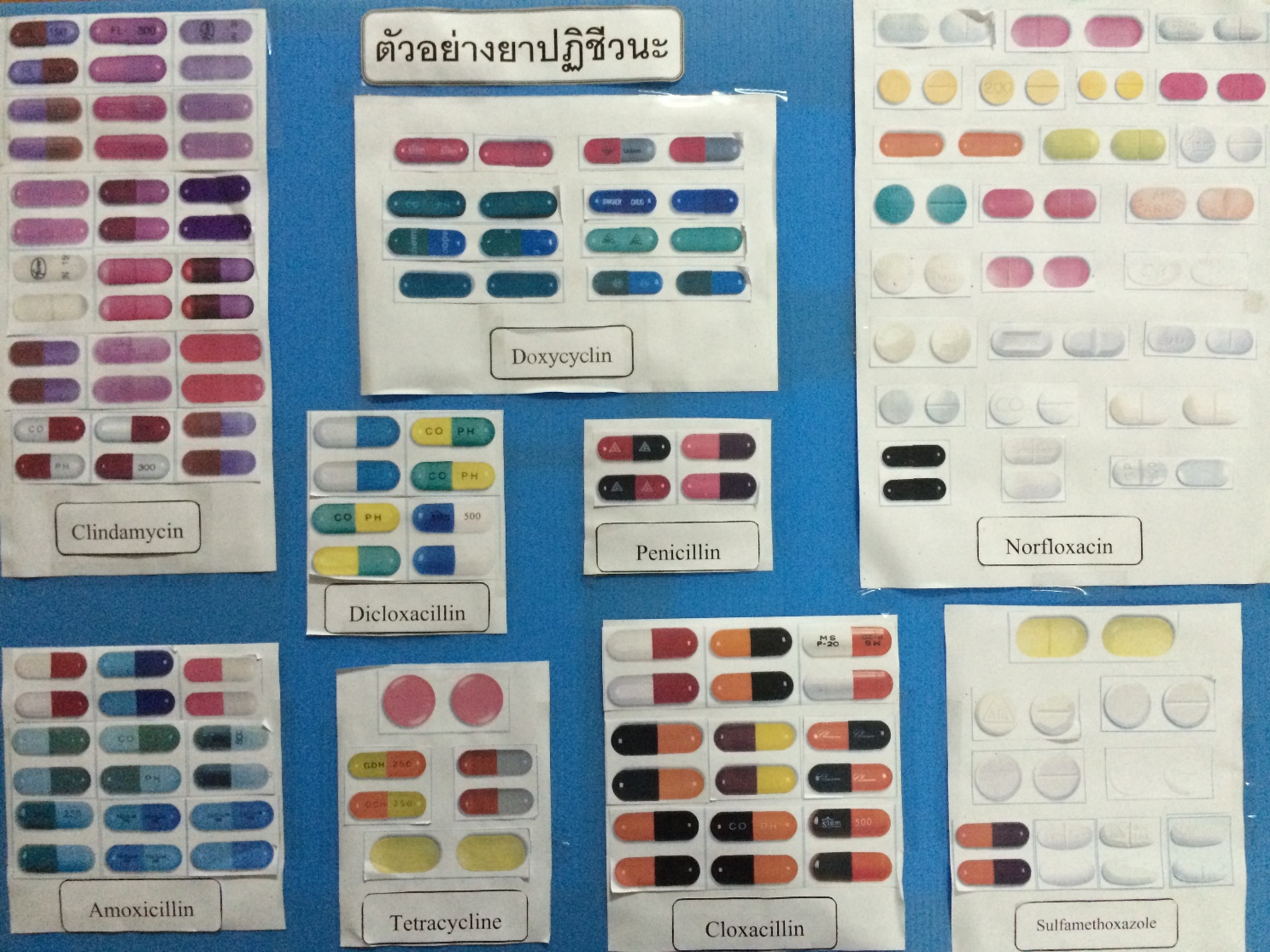


*Fig. S1. Drug Card Used During Semi-Structured Patient Interviews.*

Source: Drug card produced by study staff.

*Note*. For practical reasons, the pictures were extracted and carried and presented in an A4 folder.

# Interview Guides

Note: Sub-questions (e.g. 7a, &b) are probes depending on initial response of the interviewee.

## Patient

#### Objective:

- To shed light on why patients consume antibiotics in these settings and getting their feedback regarding their experience with the CRP test

#### Introduction

<Introduction to research following “Procedure” section of Informed Consent Form>

*Thank you for agreeing to participate in this study. My objective is to learn from you about your healthcare access and medicine use. In the questions that I am going to ask you, there are no right or wrong answers. I would like to know your personal views, experiences, and observations in order to better understand your life.*ก่อนอื่นต้องขอขอบคุณ ที่ตกลงเข้าร่วมตอบแบบสอบถามเพื่อการทำวิจัยในครั้งนี้กับเรานะคะ จุดประสงค์ของพวกเราคือเราต้องการเรียนรู้เกี่ยวกับการเข้าถึงบริการทางสุขภาพ และการใช้ยาในฐานะผู้ป่วยค่ะ ในแบบสอบถามที่เราจะใช้กับคุณชุดนี้ จะไม่มีคำตอบที่ถูกหรือผิด เราแค่ต้องการรับรู้เกี่ยวกับความคิดเห็น ประสบการณ์ และการสังเกตการณ์ของคุณเกี่ยวกับบริการทางสุขภาพเท่านั้นเองค่ะ เพื่อเราจะได้สามารถทำความเข้าใจเกี่ยวกับชีวิตของคุณได้ดีมากยิ่งขึ้นค่ะ

#### Part I – About Yourself

*Let us start with a few questions about yourself. ขอเริ่มที่ข้อมูลส่วนตัวนะคะ*

Guiding question: Who is my informant?

1. How old are you? อายุเท่าไหร่แล้วคะ
2. What is your level of education? การศึกษาสูงสุดระดับไหนคะ
3. What is your main occupation? อาชีพของคุณคืออะไรคะ
4. How large is your household? Are you the household head? ขนาดของครอบครัวที่อาศัยอยู่ด้วยกันมีสมาชิกทั้งหมดกี่คนคะ คุณเป็นหัวหน้าครอบครัวหรือไม่คะ
5. Do you identify yourself with a specific social or religious group within your community? คุณมีส่วนเกี่ยวข้องกับกลุ่มทางสังคมหรือศาสนาในชุมชนของคุณหรือไม่คะ
6. Have you lived somewhere else before? When did you move here? คุณเคยอาศัยอยู่ที่ไหนนอกเหนือจากที่อยู่ปัจจุบันหรือไม่คะ ถ้าใช่ ที่ไหนและเมื่อไหร่คะ

#### Part II – Healthcare-Seeking Behaviour

*First, I would be happy if you could tell me more about the healthcare in your household and your community. เริ่มต้นคำถามแรก อยากให้คุณช่วยพูดถึงการบริการทางด้านสุขภาพในชุมชนที่ครอบครัวของคุณใช้บริการอยู่ค่ะ*

Guiding question: How do people behave when they get ill? ที่บ้านคุณทำอย่างไรคะเวลาที่มีใครป่วย

1. You recently visited the health centre because of a fever [Guardian: you child’s fever]. ต่อไปเป็นคำถามเกี่ยวกับอาการป่วยเป็นไข้ครั้งล่าสุดของคนไข้นะคะ
   1. What was the illness? [PROBE: did your own first diagnosis differ from what you later learned? Did the different diagnoses influence your behaviour?] ไม่สบายเป็นอะไรคะ การวินิจฉัยโรคด้วยตนเองของคุณกับสิ่งที่คุณหมอวินิจฉัยให้เหมือนกันหรือไม่คะ เมื่อทราบถึงอาการป่วยที่แท้จริงจากคุณหมอแล้ว มีผลอะไรต่อพฤติกรรมของคุณบ้างมั้ยคะ
   2. What was the process of getting treatment? Please be as specific as possible, step by step. การรักษาเป็นอย่างไรคะ ช่วยอธิบายอย่างละเอียดทีละขั้นตอนตั้งแต่เริ่มมีอาการป่วยเลยค่ะ
   3. Who was involved in the decisions about what to do during the illness? มีใครมีส่วนร่วมกับการตัดสินใจของคุณเกี่ยวกับการรักษามั้ยคะ
   4. Did you take or receive any medicine or treatment at each of these steps? Do you know which these were? [point them out on drug card] คุณได้ใช้ยาในการรักษาครั้งนี้มั้ยคะ ทราบมั้ยคะว่าเป็นตัวยาตัวไหนบ้าง
   5. [if medicines taken] How did you get these medicines? [e.g. loose in bag from pharmacist, stored at home, etc.] คุณได้ยาเหล่านี้มาจากไหนคะ (ร้ายขายยา มีอยู่ในบ้าน ฯลฯ)
   6. [if medicines taken] Can you remember what you were told about how to take the medicine, and did you take them precisely in that way? [e.g. how often, for how long, how many, with or without food] คุณจำได้มั้ยคะว่าคุณใช้ยาเหล่านี้อย่างไร (ในปริมาณเท่าไหร่ ใช้อยู่นานเท่าไหร่ รับประทานกับอาหารหรือไม่) คุณสามารถปฏิบัติตามคำแนะนำการใช้ยาของคุณหมอ หรือฉลากยาได้อย่างเคร่งครัดหรือไม่คะ
2. Other than the fever that you just described, can you think of any another illness of yourself or somebody close to you, and explain to me the process as before? Was it in any way different? ครั้งล่าสุดที่คุณ หรือคนใกล้ชิดป่วยเป็น ไม่ว่าจะเป็นอะไรก็ตาม คุณมีวิธีการรักษาอย่างไรคะ เหมือน หรือ แตกต่างจากการรักษาอาการเป็นไข้อย่างไร

#### Part III – The Landscape of Healthcare and Medicines

*Now I would like to move on to healthcare providers and the availability of medicines.*

*ส่วนต่อไปเป็นเรื่องเกี่ยวกับผู้ให้บริการทางด้านสุขภาพและความพร้อมใช้งานของยาค่ะ*

Guiding question: What does the landscape of healthcare providers look like from the patient perspective? คุณมีความคิดเห็นอย่างไรเกี่ยวกับ*ผู้ให้บริการทางด้านสุขภาพ*ในชุมชนคะ

1. When would you use medicines for an illness? When not? เมื่อไหร่ที่คุณตัดสินใจใช้ยาเวลาที่คุณป่วยคะ เมื่อไหร่ที่คุณจะตัดสินใจว่าจะไม่ใช้ยา
2. Do you have preferred medicine [point out on drug card]? คุณมียาที่ใช้อยู่ประจำมั้ยคะเวลาไม่สบาย
   1. Can you explain these and what they are used for? ยาอะไรคะ ใช้ในจุดประสงค์อะไร
   2. Does that include antibiotics (e.g. amoxicillin)? [PROBE: Can you explain what antibiotics are used for?] มียาปฏิชีวนะบ้างมั้ยคะ
   3. Why do you like this medicine? ทำไมถึงเลือกใช้ยาเหล่านี้คะ
   4. Where can you get this medicine? คุณสามารถหายาเหล่านี้ได้จากไหนคะ
   5. Can you explain the process of receiving and taking them in as much as detail as possible? [PROBE: What did you do with any leftovers?] กรุณาอธิบายกระบวนการตั้งแต่ก่อนตัดสินใจซื้อยาจนกระทั่งเริ่มทานยาโดยละเอียดค่ะ ยาที่เหลือใช้ทำอะไรคะ
3. What is the best treatment for fever? เวลาที่คุณเป็นไข้ วิธีการรักษาที่ดีที่สุดคืออะไรคะ
   1. Does that include antibiotics? มีการใช้ยาปฏิชีวนะมั้ยคะ
   2. Where can you get this treatment? คุณสามารถได้รับการรักษานี้ หรือยานี้ได้จากที่ไหนคะ
4. Can you tell me which health providers are available to you, and which of them you would visit for treatment? [note: this does not have to be public or private doctors. Anyone who would be giving medical advice, treatment, or medicines, e.g. NGOs, traditional healers, untrained “health workers”, etc.] เวลาที่ไม่สบาย มีที่ไหนบ้างคะที่คุณสามารถไปใช้บริการได้ และคุณเลือกใช้แห่งไหนมากที่สุด
   1. Are there certain health conditions for which some providers are better suited than others? มีอาการป่วยชนิดไหนบ้างมั้ยคะที่คุณจะเลือกไปหาหมอที่เฉพาะเจาะจงที่คิดว่าน่าจะรักษาได้ดีกว่าหมออื่นๆ
   2. Does anything prevent you from accessing medical services? มีสิ่งใดที่เป็นอุปสรรคต่อการเข้าถึงการแพทย์มั้ยคะ

#### Part IV – Public Health Services

*We’d now like to go back to your experience at the health centre during the recent fever. ต่อไปขอพูดถึงการให้บริการของสถานีอนามัยช่วงที่คุณเข้าไปพบแพทย์ด้วยอาการไข้ครั้งล่าสุดค่ะ*

Guiding question: What is the patient experience in public health facilities, including the CRP test? มีประสบการณ์อย่างไรบ้างคะในสถานบริการทางการแพทย์ เคยตรวจวัดระดับ CRP ก่อนคุณหมอจะสั่งยาหรือไม่คะ

1. For your visit at the health centre, can you please tell me:
   1. Do you remember exactly what the HCW diagnosed or explained to you? จำได้มั้ยคะว่าคุณหมอบอกว่าเป็นอะไร
   2. What treatment or advice did you receive? ได้รับคำแนะนำและการรักษาอย่างไรบ้างคะ
   3. Were you prescribed medicines? คุณหมอได้สั่งยาให้หรือเปล่าคะ
   4. What did you like or dislike about the diagnosis and prescription process? มีอะไรที่คุณชอบหรือไม่ชอบเกี่ยวกับการวินิจฉัยโรคและการสั่งจ่ายยาของคุณหมอบ้างมั้ยคะ
   5. Did you expect that you would receive drugs during the visit? คุณได้คาดหวังมั้ยคะว่าคุณจะต้องได้รับยากลับบ้านหลังจากพบคุณหมอแน่นอน
      1. Did you then actually receive them? แล้วได้ยาจริงๆ มั้ยคะ
      2. How did you feel if you did not receive the expected medication? ถ้าไม่ได้ยาอย่างที่หวังไว้ มีความรู้สึกอย่างไรคะ
   6. Did you request a certain kind of treatment or medicine? คุณได้มีการขอยาอะไรจากคุณหมอหรือไม่คะ
   7. Could you follow the advice and instructions you were given? คุณสามารถทำตามวิธีการใช้ยาและคำแนะนำอย่างเคร่งครัดมั้ยคะ
   8. Did you trust the advice you received from the health centre? คุณมีความเชื่อมั่นในคำแนะนำที่สถานบริการทางการแพทย์มีให้กับคุณมั้ยคะ
   9. Did you receive advice, treatment, or medicine anywhere else after your visit to the health centre? หากยังมีอาการป่วยอยู่ คุณได้ไปหาหมออื่นนอกจากหมอที่พบคนแรกมั้ยคะ
2. [for patients who have been located after CRP test] A point of care test was performed during your latest visit to the [*local name of*] clinic (fingerprick blood test).
   1. Can you please describe your experiences during this process? กรุณาเล่าถึงประสบการณ์การตรวจ CRP ให้ฟังหน่อยค่ะ
   2. How did you feel, being exposed to the test? For example, did you feel worried, or curious about there being another procedure? รู้สึกอย่างไรคะตอนที่คุณหมอบอกว่าต้องตรวจเลือด และรู้สึกอย่างไรกับขั้นตอนการตรวจคะ
   3. Do you remember what you were told about the result and how it was presented to you? [e.g. did you see numbers, a chart, a colour, or did anyone tell you something like a score, or “low” and “high”?] จำได้หรือไม่คะว่าผลการตรวจเลือดออกมาเป็นอย่างไร ทราบหรือไม่คะว่าผลนั้นมีความหมายว่าอย่างไร คุณหมอใช้วิธีใดในการอธิบายผลเลือดคะ (ชาร์ตสี, บอกปากเปล่า, ตัวเลข ฯลฯ)
   4. Do you feel that you were treated differently than usual because of the test? คุณคิดว่าเจ้าหน้าที่และคุณหมอปฏิบัติตัวต่อคุณแตกต่างจากปกติ เพราะเนื่องจากการได้รับการตรวจ CRP หรือไม่คะ
   5. Did the test influence the normal interaction between you and the health worker? คุณคิดว่าการตรวจครั้งนั้นมีผลต่อปฏิสัมพันธ์ของคุณกับผู้ปฏิบัติงานมั้ยคะ
   6. Did the test affect your ability to assert yourself during the consultation? ผลการตรวจมีผลต่อการแสดงความคิดเห็นของคุณระหว่างพบคุณหมอมั้ยคะ
   7. Do you prefer the consultation with the test or the previous diagnostic procedure? คุณพอใจกับการรักษาแบบอ้างผลการตรวจ CRP หรือแบบดั้งเดิมมากกว่ากันคะ
   8. Do you trust the health worker’s advice more or less if it is based on the test? คุณจะมีความเชื่อมั่นในคำแนะนำของผู้ปฏิบัติงานมากขึ้นหรือน้อยลงคะ หลังจากได้เห็นผลตรวจ CRP แล้ว [Probe: Would that be the case even if you do not get the treatment you expect?] ถ้าคุณไม่ได้รับการรักษาในแบบที่คุณต้องการ หรือได้รับยาที่คุณคิดว่าสมควรจะได้รับ คุณยังจะเชื่อมั่นในคำแนะนำของผู้ปฏิบัติงานอยู่มั้ยคะ
   9. If there were two health centres in this town, one doing CRP and one doesn’t, which one would you go to, and why? ถ้ามีสถานีอนามัยสองที่ ที่หนึ่งตรวจ CRP อีกที่หนึ่งไม่ตรวจ คุณจะเลือกไปที่ไหนคะ เพราะอะไร

#### Wrap-up

*We are coming to the end of our interview. Before we conclude, let me just ask you: นี่เป็นส่วนสุดท้ายของการสัมภาษณ์นี้นะคะ เหลืออีกเพียงสองคำถามเท่านั้นค่ะ*

1. Have I missed an aspect of healthcare and medicine that you find particularly important? เพื่อจุดประสงค์ในการพัฒนาแบบสอบถาม จึงอยากถามคุณว่าจากคำถามที่เราได้ใช้ถามคุณมาทั้งหมด คุณคิดว่าเราพลาด หรือบกพร่องข้อมูลส่วนใดเกี่ยวกับการบริการทางด้านสุขภาพและยาที่คุณเห็นว่าควรจะกล่าวถึง และเป็นประโยชน์ในการวิจัยบ้างมั้ยคะ
2. Is there anything else you would like to share with me? มีอะไรอย่างอื่นอีกมั้ยคะที่คุณหมออยากจะแบ่งปันกับเรา

*Thank you so much for educating me about yourself and your life. Your responses have been very helpful for me.* ขอบพระคุณที่แบ่งเวลามาให้ทีมวิจัยของเราในวันนี้ได้เรียนรู้เกี่ยวกับชีวิตของคุณค่ะ คำตอบของคุณเป็นประโยชน์อย่างมากของเราและหวังเป็นอย่างยิ่งว่าคุณจะมีสุขภาพแข็งแรงหลังจากนี้นะคะ

## Health Worker

#### Objective:

- To shed some light on why health workers prescribe antibiotics in these settings and getting their feedback regarding their experience with the CRP test
- To explore the attitudes of health centre staff towards the POC CRP test

#### Introduction

<Introduction to research following Informed Consent Form>

*Thank you for agreeing to participate in this study. I would like to learn from your professional experience about the everyday work of health workers. In the questions that I am going to ask you, there are no right or wrong answers. I would like to know your personal views, experiences, and observations in order to better understand your work and life.*

ก่อนอื่นต้องขอขอบคุณคุณหมอ/พยาบาลที่ตกลงเข้าร่วมตอบแบบสอบถามเพื่อการทำวิจัยในครั้งนี้กับเรานะคะ จุดประสงค์ของพวกเราคือเราต้องการเรียนรู้จากประสบการณ์เกี่ยวกับการทำงานในแต่ละวันของผู้ปฏิบัติงานในด้านสาธารณสุข ในแบบสอบถามที่เราจะใช้กับคุณหมอ/พยาบาลชุดนี้ จะไม่มีคำตอบที่ถูกหรือผิด เราแค่ต้องการรับรู้เกี่ยวกับความคิดเห็น ประสบการณ์จากการทำงานและการสังเกตการณ์ในคนไข้ของคุณหมอ/พยาบาลเท่านั้นเองค่ะ เพื่อเราจะได้สามารถทำความเข้าใจเกี่ยวกับงานและชีวิตของคุณหมอ/พยาบาลได้ดีมากยิ่งขึ้นค่ะ

#### Part I – About Yourself

*Let us start with a few questions about yourself. ขอเริ่มที่ข้อมูลส่วนตัวนะคะ*

Guiding question: Who is my informant?

1. Name ชื่อ
2. Position / Job Title ตำแหน่ง
3. Organisation องค์กร
4. Responsibilities [incl. drug prescription] หน้าที่ความรับผิดชอบ รวมถึงการสั่งจ่ายยาด้วย
5. How long have you worked here? ทำงานที่นี่มานานเท่าไหร่แล้ว
6. How have you been trained? ได้รับการศึกษาหรือการอบรมเกี่ยวกับด้านนี้มานานเท่าไหร่แล้วคะ

#### Part II – Everyday Practice

*To begin with, please let us talk about your everyday work. ต่อไปขออนุญาตพูดถึงเรื่องชีวิตการทำงานของ*คุณหมอ/พยาบาลนะคะ

Guiding question: How is everyday work structured and what constraints do health workers face? ตารางการทำงานในแต่ละวันของคุณหมอ/พยาบาลเป็นอย่างไรคะ แล้วมีข้อจำกัดหรือแรงกดดันอะไรบ้างที่ต้องเจอในแต่ละวัน

1. What are your roles and responsibilities in your work? หน้าที่และความรับผิดชอบของคุณหมอ/พยาบาลมีอะไรบ้างคะ
   1. How much time do you spend on these activities? คุณหมอ/พยาบาลใช้เวลากับกิจกรรมเหล่านี้มากเท่าไหร่คะ
   2. Which of these activities involve outpatients? ในกิจกรรมที่กล่าวมานี้มีอันไหนบ้างคะที่เกี่ยวข้องกับผู้ป่วยนอก
   3. Can you give me an idea of a typical day? ชีวิตการทำงานแต่ละวันและสัปดาห์ของคุณหมอ/พยาบาลเป็นยังงัยคะ ทำอะไรบ้างตั้งแต่เช้าจนเลิกงาน
   4. How are you balancing your private and work life? คุณหมอ/พยาบาลมีการสร้างความสมดุลย์ระหว่างงานกับชีวิตส่วนตัวอย่างไรคะ
   5. Is there anything that makes your work particularly difficult or easy? มีอะไรบ้างคะที่ทำให้งานของคุณหมอ/พยาบาลยากขึ้นหรือง่ายขึ้นในแต่ละวัน
   6. Has this changed over the past years? มีการเปลี่ยนแปลงกับสิ่งเหล่านี้ในรอบหนึ่งปีที่ผ่านมาบ้างมั้ยคะ
2. How many outpatients do you deal with on a normal day? ในแต่ละวันคุณหมอ/พยาบาลพบผู้ป่วยนอกประมาณวันละกี่คนคะ
   1. How much time do you spend with them? แต่ละคนคุณหมอใช้เวลาพบนานประมาณเท่าไหร่คะ
   2. Does that vary seasonally? มีบางครั้งที่ใช้เวลาแตกต่างไปจากนี้มากมั้ยคะ

#### Part III – Medicines and Antibiotics Prescription

*Thank you for sharing these experiences with me. I would now like to move on to the prescription of medications to patients. ขอบคุณสำหรับการแชร์ประสบการณ์กับพวกเรานะคะ ตอนนี้เราขอเคลื่อนไปในส่วนของการสั่งจ่ายยาให้กับผู้ป่วยของ*คุณหมอ/พยาบาลค่ะ

Guiding question: How are medicines and antibiotics normally being prescribed? โดยปกติแล้วมีหลักการสั่งจ่ายยาชนิดต่างๆ และยาปฏิชีวนะอย่างไร

1. What are the three most common complaints that outpatients come to you with? ปัญหาหลักสามอย่างที่ผู้ป่วยนอกเข้ามาพบคุณหมอ/พยาบาล มีเรื่องอะไรบ้างคะ
   1. How do you diagnose these patients? คุณหมอ/พยาบาลมีวิธีการอย่างไรในการวินิจฉัยผู้ป่วยเหล่านี้คะ
   2. Are any tests being carried out (by yourself or someone else) to diagnose the illnesses? นอกจากการพูดคุยสอบถามอาการแล้ว มีการใช้วิธีการอื่นเช่นการทดสอบวัดค่าต่างๆ เพื่อเป็นส่วนช่วยในการวินิจฉัยโรคมั้ยคะ
   3. How do you choose the treatment for these conditions? คุณหมอ/พยาบาลมีวิธีการอย่างไรในการเลือกวิธีการรักษาคะ
   4. What is this procedure based on? [e.g. guidelines, behaviour of senior doctors, personal experience] การดำเนินการขั้นตอนเหล่านี้ได้หลักการมาจากอะไรคะ
   5. Are there any variations in this procedure? If so, why? นอกเหนือจากนั้นแล้วมีขั้นตอนการปฏิบัติที่แตกต่างไปจากเดิมมั้ยคะ Variation from guidelines, behaviors, etc?
   6. Which drugs do you commonly prescribe or recommend for these conditions? ส่วนใหญ่คุณหมอ/พยาบาลจะสั่งหรือแนะนำยาอะไรให้กับผู้ป่วยเหล่านี้คะ
   7. What happens if some of these drugs are not available? ในเวลาที่มีปัญหายาขาดตลาด หรือสถานอนามัยไม่มีตัวยานี้ในครอบครอง คุณหมอ/พยาบาลมีวิธีการแก้ไขปัญหาอย่างไรคะ
   8. What happens if there are complications in the treatment that the patients receive? ในกรณีที่มีภาวะแทรกซ้อน หรือปัญหาเกี่ยวกับการรักษา คุณหมอ/พยาบาลหรือสถานอนามัยนี้มีวิธีแก้ไขปัญหาอย่างไรคะ
2. [Repeat question above with focus on **fever** if respondent did not already indicate this]
3. [Note: this vignette was dropped after the first test interview as it was redundant to the previous questions and its time demands undermined the unfettered interaction between interviewer and respondent] Can you please explain what you would do in the following two cases: ในส่วนต่อไปอยากขอให้คุณหมอ/พยาบาล ช่วยอธิบายให้ฟังค่ะว่า ในเหตุการณ์สมมติที่กำลังจะเกิดขึ้นนี้ คุณหมอ/พยาบาลจะทำอย่างไร
   1. CASE 1: 5-year-old child, consulting with a runny nose, dry cough, tiredness and reduced appetite. The child has a runny nose but the rest of the physical examination is normal. เหตุการณ์สมมติที่ 1: เด็กอายุห้าขวบเข้ามาปรึกษาเรื่องคัดจมูก มีอาการไอแบบแห้งๆ มีความอ่อนเพลีย และเบื่ออาหาร หลังจากตรวจสอบพบว่าเด็กมีน้ำมูกมาก แต่การตรวจสอบในส่วนที่เหลือทั้งหมดเป็นปกติ
      1. What questions would you ask the patient about his/her condition? คุณหมอ/พยาบาลจะมีคำถามอะไรที่จะถามเพิ่งเติมกับคนไข้ หรือผู้ปกครองของคนไข้คนนี้คะ
      2. How would you normally examine this type of patient? ปกติถ้ามีคนไข้ลักษณะนี้เข้ามา คุณหมอ/พยาบาลมีวิธีการตรวจสอบอาการอย่างไรคะ
      3. Would you carry out any tests or investigations? If so which ones? คุณหมอจะเลือกตรวจสอบ หรือสังเกตอาการเพิ่มเติมหรือไม่ อย่างไร
      4. What advice/recommendations/treatment would you offer/prescribe? คุณหมอจะให้คำแนะนำ หรือการรักษากับคนไข้กรณีนี้อย่างไรคะ จะมีการสั่งยาหรือไม่ อย่างไร
   2. CASE 2: Middle aged woman with a bad cough productive of green sputum for 8 days, feels short of breath and unwell, T 38˚C, tachypnoea (fast breathing) 22/mn, R sided lung crepitations/crackles เหตุการณ์สมมติที่ 2: หญิงวัยกลางคนเข้ามาพบคุณหมอ/พยาบาลด้วยอาการไออย่างรุนแรง และมีเสมหะสีเขียวมาเป็นเวลาแปดวันแล้ว มีความรู้สึกป่วยและหายใจไม่ออก นอกจากนั้นยังมีอาการหายใจเร็ว (22 ครั้ง/นาที) ปอดข้างขวามีเสียงกรอบแกรบระหว่างหายใจ อุณหภูมิร่างกายอยู่ที่ 38 องศา
      1. What questions would you ask the patient about his/her condition? คุณหมอ/พยาบาลจะมีคำถามอะไรที่จะถามเพิ่งเติมกับคนไข้ หรือผู้ปกครองของคนไข้คนนี้คะ
      2. How would you normally examine this type of patient? ปกติถ้ามีคนไข้ลักษณะนี้เข้ามา คุณหมอ/พยาบาลมีวิธีการตรวจสอบอาการอย่างไรคะ
      3. Would you carry out any tests or investigations? If so which ones? คุณหมอจะเลือกตรวจสอบ หรือสังเกตอาการเพิ่มเติมหรือไม่ อย่างไร
      4. What advice/recommendations/treatment would you offer/prescribe? คุณหมอจะให้คำแนะนำ หรือการรักษากับคนไข้กรณีนี้อย่างไรคะ จะมีการสั่งยาหรือไม่ อย่างไร
4. Do drug company representatives promote the use of certain medicines in your health centre? ตัวแทนจำหน่ายจากบริษัทยามีการโปรโมทให้ใช้ยาชนิดใดชนิดหนึ่งเป็นพิเศษในสถานีอนามัยแห่งนี้รึเปล่าคะ
5. Do patients demand certain drugs or treatments? คนไข้ส่วนใหญ่มีการขอยาชนิดใดชนิดหนึ่งเป็นพิเศษ หรือการรักษาแบบใดเป็นพิเศษรึเปล่าคะ
6. Does that depend on their specific health condition or their beliefs? การใช้ยาหรือการขอการรักษาเป็นพิเศษแบบนี้ มีสาเหตุมาจากปัญหาสุขภาพส่วนตัวของคนไข้ หรือความเชื่อส่วนตัวบ้างมั้ยคะ
7. Does that include antibiotics? มีการเจาะจงขอยาปฏิชีวนะบ้างมั้ยคะ
8. What happens if you do not prescribe them? จะเกิดอะไรขึ้นหากคนไข้ขอยาปฏิชีวนะแล้วคุณหมอไม่สั่งให้คะ
9. Does the trust between you and the patient play a role when you recommend a treatment or drug? เวลาที่คุณหมอสั่งยา หรือเจาะจงวิธีการรักษาแบบใดแบบหนึ่ง คุณหมอคิดว่าความไว้เนื้อเชื่อใจขอคนไข้ที่มีต่อคุณหมอ มีผลต่อการ**เชื่อฟัง**ของคนไข้มั้ยคะ
10. For what conditions do you prescribe antibiotics? คนไข้ต้องมีอาการเป็นอย่างไรคะ คุณหมอถึงจะสั่งยาปฏิชีวนะ
    1. Do factors other than the patient’s illness play a role when you decide whether to prescribe antibiotics or not? มีปัจจัยอะไรอย่างอื่นบ้างมั้ยคะที่มีส่วนในการตัดสินใจของคุณหมอในการสั่งจ่ายยาปฏิชีวนะ
    2. Is diagnosis a problem when making a decision to prescribe antibiotics and other medicines? การวินิจฉัยโรคเป็นปัญหาในการตัดสินใจในการสั่งจ่ายยาปฏิชีวนะหรือยาชนิดอื่นๆ ของคุณหมอมั้ยคะ
    3. What would happen if you refuse to prescribe a patient antibiotics? หากคนไข้เจาะจงขอใช้ยาปฏิชีวนะแล้วคุณหมอปฏิเสธ จะเกิดอะไรขึ้นคะ
11. Can antibiotics be a way to protect you from patient demands, ineffective treatment, or problems in diagnosing an illness? สามารถพูดได้มั้ยคะว่าการสั่งยาปฏิชีวนะเป็นวิธีการที่จะปกป้องคุณหมอจากความต้องการที่มากเกินไปของผู้ป่วย หรือการรักษาที่ไม่มีประสิทธิภาพ หรือเวลาที่คุณหมอมีปัญหาในการวินิจฉัยอาการป่วยของคนไข้
12. Are the current levels of antibiotic prescriptions in the local health centres a problem? คุณหมอคิดว่า ระดับการสั่งจ่ายยาปฏิชีวนะกำลังสร้างปัญหามั้ยคะ
13. If you had to reduce antibiotics prescriptions, what would you consider the most effective way? ถ้าคุณหมอจำเป็นต้องลดการสั่งจ่ายยาปฏิชีวนะ คุณหมอคิดว่าจะมีวิธีใดมาแทนที่การใช้ยาปฏิชีวนะที่มีประสิทธิภาพมากที่สุด

#### Part IV – Role of CRP Testing

*Thank you. I would now like to ask you a few questions about the CRP test. ขอบคุณมากค่ะในส่วนสุดท้ายนี้ อยากถามคุณหมอเกี่ยวกับการตรวจค่า CRP ในคนไข้ค่ะ*

Guiding question: How does the test fit into current practice? [note: This section would only come in after the training has commenced] การตรวจวิธีนี้มีความเหมาะสมอย่างไรในปัจจุบันคะ

1. What has your experience with the CRP test been so far? ประสบการณ์ที่ผ่านมาของคุณหมอเกี่ยวกับการตรวจค่า CRP เป็นอย่างไรบ้างคะ
2. What do you like or dislike about the CRP test? มีสิ่งใดที่คุณหมอชอบหรือไม่ชอบเกี่ยวกับการตรวจวัดค่า CRP บ้างคะ
3. Does the test change your everyday practice? การตรวจมีส่วนเปลี่ยนแปลงชีวิตประจำวันของคุณหมอหรือไม่คะ
4. Do you trust the test results? คุณหมอมีความเชื่อมั่นในผลตรวจมั้ยคะ
5. Does the test affect your work procedure? การตรวจมีผลกระทบต่อการทำงานปกติของคุณหมอมั้ยคะ
6. Does the test affect the way you interact with patients? การตรวจมีผลกระทบต่อปฏิสัมพันธ์ของคุณหมอกับคนไข้มั้ยคะ
7. Does the test help you to decide for or against antibiotics more confidently? การตรวจมีส่วนช่วยในการสร้างความมั่นใจก่อนตัดสินใจในการสั่งจ่ายยาปฏิชีวนะของคุณหมอหรือไม่คะ
8. What are the patients’ responses when you make a decision based on the test? คนไข้มีปฏิกริยาอย่างไรคะเวลาที่คุณหมอใช้ผลจากการตรวจนี้เป็นตัวบ่งชี้ว่าควรจ่ายยาปฏิชีวนะหรือไม่
9. Are there problems in prescribing antibiotics that are not solved by the CRP test? คุณหมอเคยเจอปัญหาในการสั่งจ่ายยาปฏิชีวนะที่ไม่สามารถแก้ไขได้ด้วยการตรวจสอบค่า CRP คะ

#### Wrap-up

*We are coming to the end of our interview. Before we conclude, let me just ask you: นี่เป็นส่วนสุดท้ายของการสัมภาษณ์นี้นะคะ เหลืออีกเพียงสองคำถามเท่านั้นค่ะ*

1. Have I missed an aspect of antibiotics prescription that you find particularly important? เพื่อจุดประสงค์ในการพัฒนาแบบสอบถาม จึงอยากถามคุณหมอว่าจากคำถามที่เราได้ใช้ถามคุณหมอมาทั้งหมด คุณหมอคิดว่าเราพลาด หรือบกพร่องข้อมูลส่วนใดของยาปฏิชีวนะที่คุณหมอเห็นว่าควรจะกล่าวถึง และเป็นประโยชน์ในการวิจัยบ้างมั้ยคะ
2. Is there anything else you would like to share with me? มีอะไรอย่างอื่นอีกมั้ยคะที่คุณหมออยากจะแบ่งปันกับเรา

*Thank you so much for educating me about yourself and your life. Your responses have been very helpful for me.* ขอบพระคุณคุณหมอที่แบ่งเวลามาให้ทีมวิจัยของเราในวันนี้ได้เรียนรู้เกี่ยวกับชีวิตของคุณหมอและการปฏิบัติงานค่ะ

# Focus Group Discussion Guide

Note: Owing to the purpose of testing and expanding on the themes from the semi-structured interviews, the focus group discussion guides were more open and evolved in tandem with the insights from the interviews. For example, themes that arose during the semi-structured interviews were the use of antibiotics for sore throats rather than fevers, and the internal logic that underlies this connection.

#### Objective:

- To test the themes arising from semi-structured interviews in a more diverse and dynamic discussion group environment.

#### Introduction

<Introduction to research following “Procedure” section of Informed Consent Form>

*Thank you for agreeing to participate in this study. My objective is to learn from you about your healthcare access and medicine use. In the questions that I am going to ask you, there are no right or wrong answers. I would like to know your personal views, experiences, and observations in order to better understand your life.*ก่อนอื่นต้องขอขอบคุณ ที่ตกลงเข้าร่วมตอบแบบสอบถามเพื่อการทำวิจัยในครั้งนี้กับเรานะคะ จุดประสงค์ของพวกเราคือเราต้องการเรียนรู้เกี่ยวกับการเข้าถึงบริการทางสุขภาพ และการใช้ยาในฐานะผู้ป่วยค่ะ ในแบบสอบถามที่เราจะใช้กับคุณชุดนี้ จะไม่มีคำตอบที่ถูกหรือผิด เราแค่ต้องการรับรู้เกี่ยวกับความคิดเห็น ประสบการณ์ และการสังเกตการณ์ของคุณเกี่ยวกับบริการทางสุขภาพเท่านั้นเองค่ะ เพื่อเราจะได้สามารถทำความเข้าใจเกี่ยวกับชีวิตของคุณได้ดีมากยิ่งขึ้นค่ะ

#### Part I – About Yourself

*Let us start with a few questions about yourself. ขอเริ่มที่ข้อมูลส่วนตัวนะคะ*

Guiding question: Who is my informant?

1. How old are you? อายุเท่าไหร่แล้วคะ
2. What is your level of education? การศึกษาสูงสุดระดับไหนคะ

#### Part II – Healthcare-Seeking Behaviour

*First, I would be happy if you could tell me more about the healthcare in your household and your community. เริ่มต้นคำถามแรก อยากให้คุณช่วยพูดถึงการบริการทางด้านสุขภาพในชุมชนที่ครอบครัวของคุณใช้บริการอยู่ค่ะ*

Guiding question: How do people behave when they get ill? ที่บ้านคุณทำอย่างไรคะเวลาที่มีใครป่วย

1. You recently visited the health centre because of a fever [Guardian: you child’s fever]. What was the process of getting treatment? ต่อไปเป็นคำถามเกี่ยวกับอาการป่วยเป็นไข้ครั้งล่าสุดของคนไข้นะคะ การรักษาเป็นอย่างไรคะ
2. Other than the fever that you just described, can you think of any another illness of yourself or somebody close to you, and explain to me the process as before? Was it in any way different? ครั้งล่าสุดที่คุณ หรือคนใกล้ชิดป่วยเป็น ไม่ว่าจะเป็นอะไรก็ตาม คุณมีวิธีการรักษาอย่างไรคะ เหมือน หรือ แตกต่างจากการรักษาอาการเป็นไข้อย่างไร

#### Part III – The Landscape of Healthcare and Medicines

*Now I would like to move on to healthcare providers and the availability of medicines.*

*ส่วนต่อไปเป็นเรื่องเกี่ยวกับผู้ให้บริการทางด้านสุขภาพและความพร้อมใช้งานของยาค่ะ*

Guiding question: What does the landscape of healthcare providers look like from the patient perspective? คุณมีความคิดเห็นอย่างไรเกี่ยวกับ*ผู้ให้บริการทางด้านสุขภาพ*ในชุมชนคะ

1. When would you use medicines for an illness? When not? เมื่อไหร่ที่คุณตัดสินใจใช้ยาเวลาที่คุณป่วยคะ เมื่อไหร่ที่คุณจะตัดสินใจว่าจะไม่ใช้ยา
2. What is the best treatment for fever? เวลาที่คุณเป็นไข้ วิธีการรักษาที่ดีที่สุดคืออะไรคะ
   1. Does that include antibiotics? มีการใช้ยาปฏิชีวนะมั้ยคะ
   2. Where can you get this treatment? คุณสามารถได้รับการรักษานี้ หรือยานี้ได้จากที่ไหนคะ
3. Can you tell me which health providers are available to you, and which of them you would visit for treatment? [note: this does not have to be public or private doctors. Anyone who would be giving medical advice, treatment, or medicines, e.g. NGOs, traditional healers, untrained “health workers”, etc.] เวลาที่ไม่สบาย มีที่ไหนบ้างคะที่คุณสามารถไปใช้บริการได้ และคุณเลือกใช้แห่งไหนมากที่สุด
4. Does anything prevent you from accessing medical services? มีสิ่งใดที่เป็นอุปสรรคต่อการเข้าถึงการแพทย์มั้ยคะ

#### Part IV – Public Health Services

*We’d now like to go back to your experience at the health centre during the recent fever. ต่อไปขอพูดถึงการให้บริการของสถานีอนามัยช่วงที่คุณเข้าไปพบแพทย์ด้วยอาการไข้ครั้งล่าสุดค่ะ*

Guiding question: What is the patient experience in public health facilities, including the CRP test? มีประสบการณ์อย่างไรบ้างคะในสถานบริการทางการแพทย์ เคยตรวจวัดระดับ CRP ก่อนคุณหมอจะสั่งยาหรือไม่คะ

1. Did you expect that you would receive drugs during the visit? คุณได้คาดหวังมั้ยคะว่าคุณจะต้องได้รับยากลับบ้านหลังจากพบคุณหมอแน่นอน
2. Did you receive advice, treatment, or medicine anywhere else after your visit to the health centre? หากยังมีอาการป่วยอยู่ คุณได้ไปหาหมออื่นนอกจากหมอที่พบคนแรกมั้ยคะ
3. [for patients who have been located after CRP test] A point of care test was performed during your latest visit to the [*local name of*] clinic (fingerprick blood test). Can you please describe your experiences during this process? กรุณาเล่าถึงประสบการณ์การตรวจ CRP ให้ฟังหน่อยค่ะ

#### Wrap-up

*We are coming to the end of our interview. Before we conclude, let me just ask you: นี่เป็นส่วนสุดท้ายของการสัมภาษณ์นี้นะคะ เหลืออีกเพียงสองคำถามเท่านั้นค่ะ*

1. Have I missed an aspect of healthcare and medicine that you find particularly important? เพื่อจุดประสงค์ในการพัฒนาแบบสอบถาม จึงอยากถามคุณว่าจากคำถามที่เราได้ใช้ถามคุณมาทั้งหมด คุณคิดว่าเราพลาด หรือบกพร่องข้อมูลส่วนใดเกี่ยวกับการบริการทางด้านสุขภาพและยาที่คุณเห็นว่าควรจะกล่าวถึง และเป็นประโยชน์ในการวิจัยบ้างมั้ยคะ
2. Is there anything else you would like to share with me? มีอะไรอย่างอื่นอีกมั้ยคะที่คุณหมออยากจะแบ่งปันกับเรา

*Thank you so much for educating me about yourself and your life. Your responses have been very helpful for me.* ขอบพระคุณที่แบ่งเวลามาให้ทีมวิจัยของเราในวันนี้ได้เรียนรู้เกี่ยวกับชีวิตของคุณค่ะ คำตอบของคุณเป็นประโยชน์อย่างมากของเราและหวังเป็นอย่างยิ่งว่าคุณจะมีสุขภาพแข็งแรงหลังจากนี้นะคะ
